# Polyphenolic Compounds Isolated from Marine Algae Attenuate the Replication of SARS-CoV-2 in the Host Cell through a Multi-Target Approach of 3CL^pro^ and PL^pro^

**DOI:** 10.3390/md20120786

**Published:** 2022-12-19

**Authors:** D. P. Nagahawatta, N. M. Liyanage, Jung-Geon Je, H. H. A. C. K. Jayawardhana, Thilina U. Jayawardena, Seong-Hun Jeong, Hyung-Jun Kwon, Cheol Soo Choi, You-Jin Jeon

**Affiliations:** 1 Department of Marine Life Sciences, Jeju National University, Jeju 690-756, Republic of Korea; 2 Department of Chemistry, Biochemistry and Physics, Université du Québec à Trois-Rivières, Trois-Rivières, QC G8Z 4M3, Canada; 3 Functional Biomaterial Research Center, Korea Research Institute of Bioscience and Biotechnology, Daejeon 56212, Republic of Korea; 4 Korea Mouse Metabolic Phenotyping Center, Lee Gil Ya Cancer and Diabetes Institute, Gachon University, Incheon 21999, Republic of Korea; 5 Division of Endocrinology & Metabolism, Department of Internal Medicine, Gil Medical Center, Gachon University College of Medicine, Incheon 21565, Republic of Korea

**Keywords:** SARS-CoV-2, 3CL^pro^, PL^pro^, marine natural product, molecular docking, multi-target approach

## Abstract

A global health concern has emerged as a response to the recent SARS-CoV-2 pandemic. The identification and inhibition of drug targets of SARS-CoV-2 is a decisive obligation of scientists. In addition to the cell entry mechanism, SARS-CoV-2 expresses a complicated replication mechanism that provides excellent drug targets. Papain-like protease (PL^pro^) and 3-chymotrypsin-like protease (3CL^pro^) play a vital role in polyprotein processing, producing functional non-structural proteins essential for viral replication and survival in the host cell. Moreover, PL^pro^ is employed by SARS-CoV-2 for reversing host immune responses. Therefore, if some particular compound has the potential to interfere with the proteolytic activities of 3CL^pro^ and PL^pro^ of SARS-CoV-2, it may be effective as a treatment or prophylaxis for COVID-19, reducing viral load, and reinstating innate immune responses. Thus, the present study aims to inhibit SARS-CoV-2 through 3CL^pro^ and PL^pro^ using marine natural products isolated from marine algae that contain numerous beneficial biological activities. Molecular docking analysis was utilized in the present study for the initial screening of selected natural products depending on their 3CL^pro^ and PL^pro^ structures. Based on this approach, Ishophloroglucin A (IPA), Dieckol, Eckmaxol, and Diphlorethohydroxycarmalol (DPHC) were isolated and used to perform in vitro evaluations. IPA presented remarkable inhibitory activity against interesting drug targets. Moreover, Dieckol, Eckmaxol, and DPHC also expressed significant potential as inhibitors. Finally, the results of the present study confirm the potential of IPA, Dieckol, Eckmaxol, and DPHC as inhibitors of SARS-CoV-2. To the best of our knowledge, this is the first study that assesses the use of marine natural products as a multifactorial approach against 3CL^pro^ and PL^pro^ of SARS-CoV-2.

## 1. Introduction

An unknown series of pneumonia cases were identified in December 2019 and emerged in Wuhan, Hubei province, China. According to the World Health Organization (WHO) office in China, the infected clusters were initially reported on the 31 December 2019. A novel type of coronavirus was identified by Chinese authorities on 7 January 2020, which caused a new, infectious, respiratory disease called severe acute respiratory syndrome coronavirus 2 (SARS-CoV-2). The identified coronavirus expressed significant differences compared to other respiratory pathogens, such as severe acute respiratory coronavirus (SARS-CoV) and Middle East respiratory syndrome coronavirus (MERS-CoV), influenza, adenovirus, and avian influenza. The origin of SARS-CoV-2 remains unclear, but RaTG13, the coronavirus isolated from bats, expressed a genetic similarity close to SARS-CoV-2. Therefore, bats are considered the origin of this disease [1]. However, the transmission mechanism of the virus from bats to human beings remains unclear.

Coronaviruses that belong to the *Coronaviridae* family are enveloped, non-segmented, positive-sense, single-stranded RNA viruses in the *Nidovirales* order [2]. The abovementioned viruses infect humans and other mammals to a considerable extent. Furthermore, Chinese horseshoe bats were identified as natural reservoir hosts for SARS-CoV [3]. SARS-CoV was therefore controlled using conventional methods such as travel restrictions and the isolation of patients. The infection mechanism of SARS-CoV-2 is not yet fully understood, including the reasons why human beings are the principal hosts of the virus and how it escapes their innate immune systems. Moreover, the interaction between the human Toll-like receptor (TLR), viral antigens, the mechanism of pro-inflammatory cytokine production, and its effect on important human organs, are not yet fully understood. However, the viral entry mechanism has been identified in the literature, and it invades the human body through the respiratory system using respiratory droplets via sneezing and coughing [4]. SARS-CoV-2 consists of a protein capsid covered by glycoprotein with anchored spike proteins. These spike proteins initiate viral entry into the target cells. The entry of SARS-CoV-2 into the host cell is an important factor required to determine the infectivity and pathogenesis of the virus [5]. Therefore, it is determined to be a key target for host immune monitoring and human intervention strategies [6]. The SARS-CoV-2 spike protein initially binds to the cell surface receptor called angiotensin-converting enzyme 2 (ACE-2), and this is known as viral attachment; subsequently, it enters the endosome and, finally, the viral membrane fuses with the lysosomal membrane [7]. However, the continuous mutation of the SARS-COV-2 spike protein has made the development of an antiviral drug for viral infections using a spike protein inhibition strategy challenging. Therefore, in the present study, we identified that viral replication occurring in the host cell is the most successful way to control the viral load in the host.

Coronaviruses contain RNA viral genomes that are 26 to 32 kb in length. The newly sequenced SARS-CoV-2 genome was submitted to the NCBI genome database under accession number NC_045512.2, and the size was ~29.9 kb [8]. SARS-CoV-2 consists of 13 to 15 open reading frames (ORFs), including 12 functioning ORFs. The ORFs are arranged as nucleocapsid proteins. When considering the whole genome of SARS-CoV-2, it encodes for polyproteins that consist of ~7096 residues. It contains many structural and non-structural proteins (NSPs) as well as ORF1a and ORF1b, which encode for non-structural proteins and are mainly responsible for the nucleotide content of the genome. ORFs 1a and 1b encode the polyproteins pp1a and pp1b, respectively, and gene 1b employs the ribosomal frameshift mechanism to encode pp1ab. The virally encoded proteases cleave these polyproteins and produce 16 NSPs and the rest of the genomes responsible for the creation of structural proteins. These proteins play a pivotal role in viral-entry fusion, replication, and survival in host cells. Thus, these gene products are considered the main drug or vaccine targets [9]. Polyprotein processing is mainly conducted by the 3-chymotrypsin-like protease (3CL^pro^) and papain-like protease (PL^pro^). The polyprotein is cleaved at 11 distinct sites by 3CL^pro^. This leads to the production of NSPs that are important in the process of viral replication [10]. A key role is played by 3CL^pro^ in SARS-CoV-2 replication in the host cell. According to the previous studies, high-throughput studies and structure-based activity analysis confirmed the value of the potential inhibitors of the activity of 3CL^pro^ against SARS-CoV and MERS-CoV, which successfully inhibited virus replication activity [11,12,13]. Therefore, the 3CL^pro^ of SARS-CoV-2 is considered a potential drug candidate. The PL^pro^ of SARS-CoV and SARS-CoV-2 expresses an 83% sequence identity and diverges from MERS-CoV. However, the host substrate preference of PL^pro^ is the difference between these two strains. Furthermore, the PL^pro^ of SARS-CoV-2 cleaves the ubiquitin-like interferon-stimulated gene 15 protein (ISG15), and PL^pro^ of SARS-CoV predominantly targets the ubiquitin chain [14]. ISG15 regulates various cellular signaling pathways and host immune responses. Therefore, 3CL^pro^ and PL^pro^ are identified as potential drug targets for the inhibition of SARS-CoV-2.

Marine algae confront extreme environmental conditions, and their metabolism consists of a biochemical process that absorbs nutrients and converts them into materials that are important for survival in these specific environmental conditions [15]. These accumulated defense metabolites express an elevated potential to develop novel therapeutic agents [16]. Among these secondary metabolites, phlorotannins, such as polyphenolic compounds, have been identified in the research as potential antiviral agents for various types of viruses, including enveloped [17,18,19] and non-enveloped viruses [20,21,22], which exert their antiviral activity through inhibiting vital viral proteins.

Thus, the present study aims to inhibit SARS-CoV-2 through 3CL^pro^ and PL^pro^ using marine natural products isolated from marine algae. Molecular docking was utilized for the initial screening of selected natural products (MNPs) based on the 3CL^pro^ and PL^pro^ protein structures. Moreover, the resulting compounds were isolated and used for biological assays for further confirmation of the inhibition activity. In the present study, we utilize an in vitro assay kit and introduce a simplified method to determine the inhibitory activity of the compounds using a cell-based assay. To the best of our knowledge, this is the first study that assesses the function of marine natural products in relation to 3CL^pro^ and PL^pro^ of SARS-CoV-2 as a multi-target approach.

## 2. Results

### 2.1. Structure of 3CL^pro^ and PL^pro^ Receptor Proteins and Ligands

The previously resolved X-ray crystallography of SARS-CoV-2 3CL^pro^ and PL^pro^ at the high resolutions of 2.16 Å and 2.59 Å was obtained from PDB (3CL^pro^ PDB ID 6LU7 and PL^pro^ PDB ID 7CMD) in a complex with the N3 inhibitor (ID PRD_002214) and GRO0617, respectively. The total structure weight values of 3CL^pro^ and PL^pro^ were 34.4 kDa and 145.97 kDa., respectively. The N3 inhibitor was bound to the present structure by conventional hydrogen bonds with PHE140, GLY143, HIS164, GLU166, GLN189, and THR189 residues; carbon–hydrogen bonds with ASN142, Met165, and HIS172; and alkyl bonds with HIS41, MET49, LEU167, PRO168, and ALA191. The radius of the prepared binding-site sphere was 13.82 Å and HIS41, MET49, PHE140, ASN142, GLY143, HIS164, MET165, GLU166, LEU167, PRO168, HIS 172, GLN189, THR190, and ALA191. The prepared 3CL^pro^ was superimposed with the original 3CL^pro^ available in PBD PyMOL, and the calculated RMSD value was 0.185 (Supplementary Figure S1). The GRL0617 was bound with the PL^pro^ enzyme using conventional hydrogen bonds with ASP164 and GLN269, residues, a carbon–hydrogen bond with a TYR268 residue, and alkyl bonds with LEU162, PRO247, PRO248, TYR264, and TYR273 residues. The ligand-binding site was defined using a binding-site sphere with a 15.9 Å radius, including PRO247, PRO248, LEU162, ASP164, TYR268, GLU269, and TYR273. The prepared PL^pro^ was superimposed with the original PL^pro^ available in PBD using PyMOL, and the calculated RMSD value was 0.235 (Supplementary Figure S2). A total of 16 ligands were prepared using DS “Prepare ligand”, and the most stable ligand conformation was used to perform molecular docking.

### 2.2. Molecular Docking

Molecular docking was performed between all ligands and 3CL^pro^ or PL^pro^ receptor proteins separately. The corresponding dock scores are summarized in Table 1 and Table 2, respectively. According to the results obtained for flexible docking, binding energy, and the DS visualizer, IPA, DPHC, Dieckol, and Eckmaxol were selected for further studies.

#### 2.2.1. The Binding Affinity of Ligands with the 3CL^pro^ Enzyme

IPA was bound to the binding site of 3CL^pro^ through THR26, ASN119, PHE140, ASN142, GLY143, PRO168, and THR190 residues via conventional hydrogen bonds with the lengths of 2.35 Å, 2.30 Å, 1.92 Å, 1.99 Å, 2.93 Å, 2.96 Å, and 2.31 Å, respectively, and GLU166 produced 3 hydrogen bonds with the lengths of 1.94 Å, 1.97 Å, and 1.94 Å. Furthermore, a one pi-sulfur bond with MET49 of a 5.08 Å length and a pi-alkyl bond with LEU141 of a 5.43 Å length can be observed (Supplementary Figure S3).

Dieckol was bound to 3CL^pro^ through four conventional hydrogen bonds with PHE140, ASN142, GLU166, PRO168, and ARG188, and the lengths of the bonds were 2.91 Å, 2.88 Å, 3.97 Å, 2.01 Å, and 2.21 Å, respectively. Furthermore, there were 3 pi–pi T-shaped bonds with HIS41, LEU141, and HIS164 residues, and the lengths of each bond were 6.87 Å, 4.19 Å, and 5.33 Å, respectively, and 4 pi-anion bonds. Among these bonds, binding occurred between the length of 5.28 Å to CYS145, 5.33 Å length to HIS164, 4.41 Å length to MET165, and 5.64 Å, 5.22 Å lengths to the GLU166 residue, and finally, two pi-alkyl bonds to an MET165 residue. The lengths of each bond were 4.67 Å and 5.09 Å (Supplementary Figure S4).

Eckmaxol was bound to 3CL^pro^ using ASN142, GLY143, SER144, HIS164, GLU166, ASP187, and GLN189 residues via conventional hydrogen bonds with 2.34 Å, 1.78 Å, 4.17 Å, 2.09 Å, 2.82 Å, 2.10 Å, and 1.88 Å lengths, respectively. Furthermore, there were 4 carbon-hydrogen bonds, PHE140, LEU141, MET165, and LEU167, with lengths of 2.47 Å, 2.58 Å, and 2.7 Å, respectively, and 2 pi-alkyl bonds with MET49 and CYS145 residues with lengths of 4.62 Å and 4.77 Å (Supplementary Figure S5).

DPHC was stabilized in the active site of 3CL^pro^ through three conventional hydrogen bonds with CYS44, CYS 145, and GLU166; five carbon–hydrogen bonds with THR24, THR 25, MET165, ASN142, and ASN143; and one salt bridge with HIS163. The bond lengths of conventional hydrogen bonds were 1.89 Å–2.3 Å, the lengths of the carbon–hydrogen bonds were 2.56 Å–2.67 Å; and the length of the salt bridge was 5.82 Å (Supplementary Figure S6).

#### 2.2.2. The Binding Affinity of Ligands with the PL^pro^ Enzyme

IPA was bound to PL^pro^ by six conventional hydrogen bonds through ASP164, GLN250, TYR267, and TYR273 with lengths of 2.33 Å, 1.86 Å, 1.91 Å, and 1.91 Å, respectively. IPA made produced conventional hydrogen bonds via ASN267 with 2.24 Å and 2.44 Å lengths. Furthermore, three pi-alkyl bonds between IPA and PL^pro^ were produced using PRO247 and ALA249 with 4.69 Å, 5.33 Å, and 4.88 Å lengths; one pi-sulfur bond with an MET208 residue with a length of 5.98 Å; and one pi-anion bond using ASP164 with a length of 3.24 Å (Supplementary Figure S7).

Dieckol created 3 conventional hydrogen bonds with PL^pro^ using GLY163, TYR273, and THR302 residues and the bond lengths were 1.91 Å, 2.18 Å, and 2.06 Å. Furthermore, PRO247 and SER245 produced a carbon–hydrogen bond with 2.51 Å and 2.54 Å lengths. Moreover, PRO2478 produced a pi-alkyl bond with a 5.50 Å length. ARG166 created a salt bridge with a length of 1.75 Å and ASP164 bound to Dieckol via three pi-anions with 4.87 Å, 4.0 Å, and 3.90 Å lengths. ALA246 produced two amide-pi-staked bonds with lengths of 5.50 Å and 5.37 Å. The THR301 residue created a 2.88 Å length pi-lone pair using Dieckol (Supplementary Figure S8).

Eckmaxol was bound to PL^pro^ using ARG166, TYR273, and ASP302 residues via conventional hydrogen bonds with 2.01 Å, 2.41 Å, and 2.76 Å lengths, respectively. Furthermore, there were two carbon–hydrogen bonds between Eckmaxol and PRO248 and TYR248 with lengths of 2.62 Å and 2.91 Å, respectively. PRO247 produced two pi-alkyl bonds using Eckmaxol with lengths of 5.00 Å and 4.98 Å, and POR248 and ARG166 created an additional pi-alkyl bond with the lengths of 5.36 Å and 5.43 Å, respectively (Supplementary Figure S9).

DPHC produced two conventional hydrogen bonds with PL^pro^ via two conventional hydrogen bonds with GLN270 and THR302 (1.94 Å and 1.92 Å); one carbon–hydrogen bond with PRO249 (2.22 Å); and three weak bonds, including an active charge with LYS158, a pi-cation with ASP165, and pi-anion with ARG167 (5.68–5.69 Å) (Supplementary Figure S10).

### 2.3. In Vitro Inhibitory Potential of MNPs

**Figure 1:**
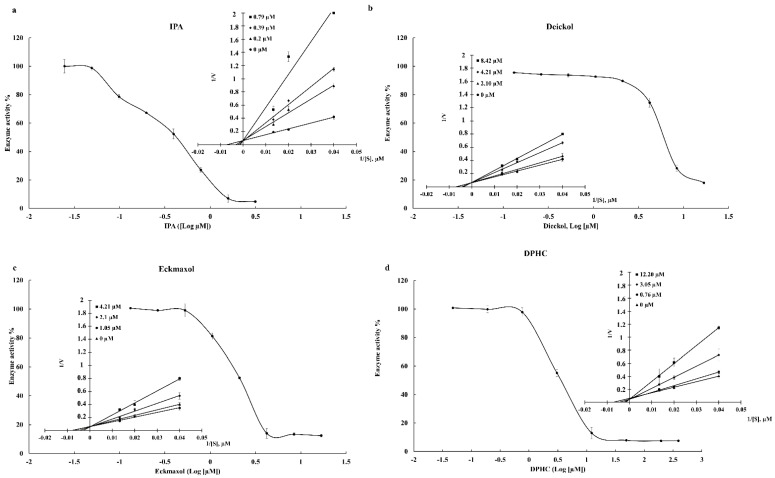
Inhibitory effects of (**a**) Ishophloroglucin A (IPA), (**b**) Dieckol, (**c**) Eckmaxol, and (**d**) Diphlorethohydroxycarmalol (DPHC) on the activity of SARS-CoV-2 3CLpro and enzyme kinetic activity.

**Figure 2:**
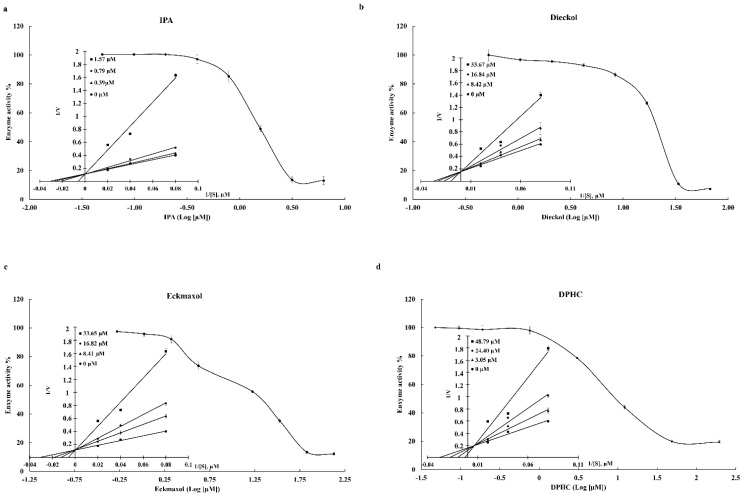
Inhibitory effects of (**a**) Ishophloroglucin A (IPA), (**b**) Dieckol, (**c**) Eckmaxol, and (**d**) Diphlorethohydroxycarmalol (DPHC) on the activity of SARS-CoV-2 PL^pro^ and enzyme kinetic activity.

The inhibition ability of IPA, DPHC, Dieckol, and Eckmaxol was evaluated using an in vitro inhibition assay kit of 3CL^pro^ and PL^pro^. The broad-spectrum antiviral medication GC376 was used as a positive control against 3CL^pro^ [23], and GRL0617 was used as the positive control against PL^pro^ [24]. The results are summarized in Table 3. As shown in the results, IPA expresses remarkable inhibition activity against 3CL^pro^ and PL^pro^. Moreover, DPHC, Dieckol, and Eckmaxol show significant inhibitory activity against the proteolytic activity of 3CL^pro^ and PL^pro^. This significant and dose-dependent inhibition is clearly presented in Figure 1 and Figure 2.

#### Lineweaver–Burk Plot

The inhibition data were plotted in the Lineweaver–Burk plot, which determines the mode of inhibition. The V_max_ of all compounds was unchanged, and K_m_ was increased with the inhibitor concentration. This revealed that IPA, DPHC, Dieckol, and Eckmaxol inhibited 3CL^pro^ and PL^pro^ as competitive inhibitors (Figure 1 and Figure 2).

### 2.4. Cell-Based Inhibition of 3CL^pro^ and PL^pro^ Using MNPs

A cell-based inhibition assay for 3CL^pro^ and PL^pro^ was subsequently performed to further confirm the molecular docking activity and in vitro assay kit results. The selected MNP concentrations were non-toxic to the Vero E-6 cells. All MNPs were dissolved in DMSO and diluted using 1X phosphate-buffered saline. The final DMSO concentration in the highest diluted MNP concentration was less than 1%.

**Figure 3:**
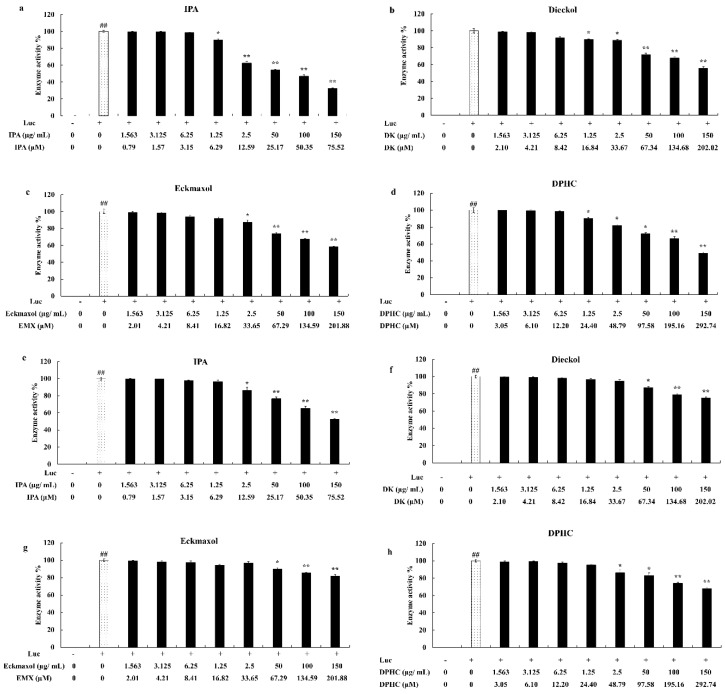
Cell-based 3CL^pro^ inhibitory activities of (**a**) Ishophloroglucin A (IPA), (**b**) Dieckol, (**c**) Eckmaxol, and (**d**) Diphlorethohydroxycarmalol (DPHC), and cell-based PL^pro^ inhibitory activities of (**e**) Ishophloroglucin A (IPA), (**f**) Dieckol, (**g**) Eckmaxol, and (**h**) Diphlorethohydroxycarmalol (DPHC). Triplicate experiments were used to evaluate the data and the mean value is expressed with ±SD. * *p* < 0.05, ** *p* < 0.01, against PM-treated group or ## *p* < 0.01, against control (ANOVA, Duncan’s multiple range test).

All MNPs successfully inhibited the proteolytic activity of 3CL^pro^ and PL^pro^ in a dose-dependent manner. IPA exhibited significant and dose-dependent inhibitory activity against 3CL^pro^ and PL^pro^ at 6.29–75.52 µM and 12.59–75.52 µM, respectively. Furthermore, IPA showed the greatest inhibitory activity against the proteolytic activity of both 3CL^pro^ and PL^pro^ compared to other MNPs. Moreover, DPHC, Eckmaxol, and Dieckol also showed significant and dose-dependent inhibitory activities (Figure 3).

### 2.5. CPE Reduction Effect

**Figure 4:**
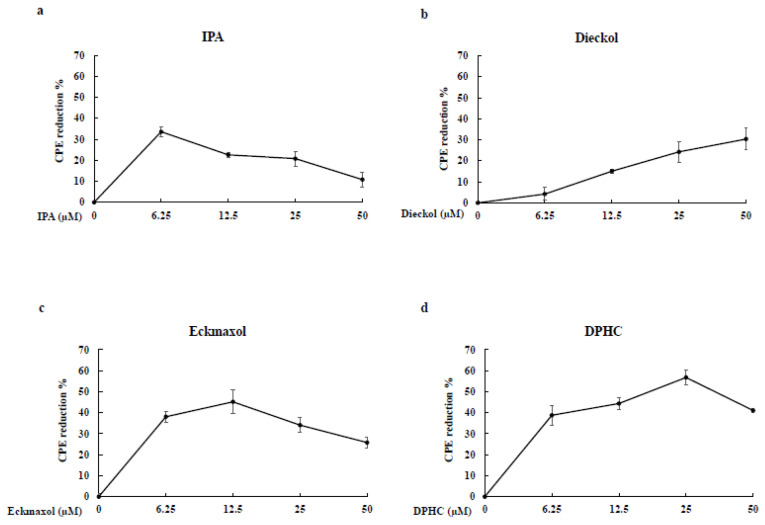
Inhibitory activity of cytopathic effect induced by SARS-CoV-2: (**a**) Ishophloroglucin A (IPA), (**b**) Dieckol, (**c**) Eckmaxol, and (**d**) Diphlorethohydroxycarmalol (DPHC).

According to the CPE reduction assay results, IPA, DPHC, and Eckmaxol expressed great CPE reduction activity at 6.25 µM against SARS-CoV-2 compared to Dieckol. The greatest CPE reduction activity was exhibited by DPHC at 25 µM. Furthermore, Dieckol presented a dose-dependent inhibitory activity against SARS-COV-2 (Figure 4).

## 3. Discussion

The emergence of COVID-19 in December 2019 resulted in a pandemic that was responsible for millions of deaths. The health sector developed several vaccines and attempted to repurpose US Food and Drug Administration (FDA)-approved drugs. However, these attempts were not sufficient to eliminate COVID-19, and most vaccines failed due to the occurrence of viral mutations. This emphasized the development of an antiviral agent to be used against SARS-CoV-2. In the various studies that provide insights into the development of an antiviral agent for SARS-CoV-2, such as computational studies, in vitro, in vivo, and clinical trials can be observed. However, the authors of the present study attempted to develop an antiviral agent for several drug targets, and this multi-target approach was more successful than inhibiting individual drug targets.

The present study used Discovery Studio software to perform the initial screening. The “Prepare protein” tool of the software successfully prepared the structures of 3CL^pro^ and PL^pro^ using 6LU7 and 7CMD structures of PDB. The “Prepare protein” tool was utilized to solve the most common problems, such as removing alternate conformations, removing heteroatoms, hydrogen additions, and correcting missing or incorrectly specified residues. The energy minimization of target receptor proteins was performed using the “Protein minimization” tool. DS provides three options to prepare the binding site of the receptor protein: “based on the PDB site records”, “from receptor cavities”, and “form current selection”. The present study used the final tool based on the available ligands of the crystal structures and previously published data. The results of the molecular docking reveal that compounds IPA, DPHC, Dieckol, and Eckmaxol showed the greatest binding affinity to the active sites of 3CL^pro^ and PL^pro^. Therefore, these compounds were selected for further analysis.

The current study used an in vitro assay kit that was specially designed to determine 3CL^pro^ and PL^pro^ inhibitions. The isolated polyphenolic compounds were utilized to determine inhibitory activity against 3CL^pro^ and PL^pro^. All of the tested compounds exhibited dose-dependent inhibitory activity against 3CL^pro^ and PL^pro^. GC376 (IC_50_ 0.4231 µM) and GRL0617 (IC_50_ 1.5 µM) were used as positive controls against 3CL^pro^ and PL^pro^, respectively [14,25,26]. Among these compounds, IPA presented remarkable inhibitory activity against both proteases 3CL^pro^ and PL^pro^ with IC_50_ values of 0.4814 and 1.4048 µM, respectively. Furthermore, DPHC, Dieckol, and Eckmaxol also exhibited significant inhibitory activity. These results strengthen the in silico outcomes. The binding affinity of these compounds was evaluated based on the active site of the protease enzymes. Thus, a Lineweaver–Burk plot was created to determine the inhibitory patterns, according to the enzyme kinetic results, by increasing K_m_ and constant V_max_ with the increasing compound concentration. This confirmed that all compounds were competitive inhibitors. These results also confirm the in silico results that all the compounds are bound to the active site of the protease enzymes.

3CL^pro^ and PL^pro^ inhibitions were evaluated further using a cell-based inhibitory assay. This method did not require protein purification and was close to the natural physiological state. Thus, this method can be successfully used to strengthen the previous results. The in-frame construction of 3CL^pro^ or PL^pro^ with the substrate (the peptide sequence that contained a cleavage site) and firefly luciferase gene was designed as the plasmid transfected into the Vero E-9 cells. Normally, this method should be performed as a co-transfection process using the indicated vector. However, it can affect the accuracy of the final results. Therefore, we used a vector that contained both renilla and firefly luciferases. Furthermore, renilla luciferase was expressed independently from the protease gene or firefly luciferase. Thus, the luminescence from the firefly luciferase can be normalized using renilla luciferase expression. The luciferase protein that is bound to another protein with a value greater than 33 kDa remarkably decreased the luciferase activity [27]. Thus, a peptide sequence that contains a cleavage site for 3CL^pro^ or PL^pro^ was used for binding the protease enzyme with the firefly luciferase. Thus, the inhibitory activity of MPNs downregulated the luciferase activity [28] and did not interfere with renilla luciferase activity. These results also exhibit the significant inhibitory activity of MNPs against 3CL^pro^ and PL^pro^. However, the IC_50_ values of the results present a significant difference compared to the in vitro assay kit. The permeability of the compounds and cell membrane could be the reason for the results we obtained [29]. Furthermore, the results confirm that IPA is the most efficient inhibitor of both proteases. SARS-CoV-2 can affect cells in numerous organs and systems present in the human body and mostly infected the upper and lower respiratory tracts. Among them, the lungs are the most infected organ due to the presence of ACE-2 receptors [30]. Therefore, the CPE of the virus on these tissues and the reduction in CPE are important factors. The CPE reduction evaluation is a commonly used assay format for screening antiviral agents. The cell death caused by viral infection was measured using this assay [31]. The CPE reduction effect of all compounds against the virus was evaluated, and among them, IPA, DPHC, and Eckmaxol exhibited remarkable CPE reduction activity. However, further studies are required to reveal and confirm the exact behavior of these compounds in an in vivo model.

## 4. Conclusions

In this study, we, for the first time, reported the potential of polyphenolic compounds isolated from brown marine algae as an inhibitor against SARS-CoV-2 using two main drug targets that play a pivotal role in viral replication and survival in the host cell. The binding affinity of the selected molecules was evaluated using a molecular docking study. The in silico results reveal interesting molecules that have the potential to bind with the active site of each protein. The inhibition activities of isolated compounds against 3CL^pro^ and PL^pro^ were assessed with a molecular in vitro biological assay kit and cell-based inhibition. The results show that all four compounds significantly downregulate the proteolytic activity of 3CL^pro^, and PL^pro^ is significantly downregulated. Additionally, the potential of IPA as the most potent inhibitor through the multiple approaches used against SARS-CoV-2 was identified. The CPE reduction assay reinforced these outcomes. However, according to the CPE reduction assay results, DPHC and Eckmaxol present greater activities than IPA. Thus, further in vivo and clinical studies are required to confirm the behavior of these compounds.

## 5. Methods and Materials

### 5.1. Chemicals and Reagents

Dimethylsulfoxide (DMSO) and all the organic solvents (HPLC grade) used in the experiments were purchased from Sigma-Aldrich (St Louis, MO, USA). The in vitro inhibition assay kits for 3CL^pro^ and PL^pro^ were purchased from the AMSBIO company (Madrid, Spain). Dulbecco’s modified Eagle’s medium (DMEM) was purchased from Gibco/BRL (Burlington, ON, Canada)), 10% fetal bovine serum (FBS) and 5% penicillin/streptomycin were purchased from WELGENE (Gyeongsan, Korea), and 3-(4,5-Dimethylthiazol-2-yl)-2,5-diphenyltetrazolium bromide (MTT) was purchased from Sigma Aldrich (St. Lois, MO, USA). The Nano-Glo^®^ Dual-Luciferase^®^ Reporter Assay System was purchased from Promega (Madison, Wisconsin, USA), the genes were purchased from Sino Biological (Beijing, China), and the pcDNA3 RLUC POLIRES FLUC vector was purchased from addgene (Watertown, Massachusetts, USA). The Vero E6 cell line was purchased from the American Type Culture Collection (ATCC, Manassas, VA, USA).

### 5.2. Preparation of Receptors

The protein Data Bank (PDB) (http://www.pdb.org, accessed on 20 October 2020) was used to obtain the crystal structures of 3CL^pro^ and PL^pro^ under PDB ID: 6LU7 and 7CMD, respectively. The molecular docking studies were conducted using Discovery Studio (DS-Client v18.1.100.18065). Briefly, the crystal structure of each protein was downloaded from PDB. The water molecules and heteroatoms were removed and the “Clean protein” tool was used to correct any minor problems, such as missing-side chain atoms, which were added in an extended confirmation. Then, the “Prepare protein” tool was utilized for further preparations of the receptor protein, and the energy was minimized using the “Protein minimization” tool. The crystal structure of 3CL^pro^ was available in the PDB as a complex with an inhibitor called the Michael-acceptor or N3 inhibitor. The binding site of 3CL^pro^ was determined based on the abovementioned inhibitor and previous studies [32]. PL^pro^ was available in the PDB as a complex with a GRL0617 inhibitor. Therefore, the binding site of PL^pro^ was determined using GRL0617 and previous studies [24]. Briefly, the binding site was prepared as a sphere in the ligand-binding site of the crystal structure, and the prepared binding sites of the target proteins were identified by specifying a sphere of a given radius located in the active site. The geometric center of the ligand in the crystal structure was used as the center of the sphere. A Python-enhanced molecular graphics tool (PyMOL, version 2.4.1) was used to calculate the root-mean-square deviation of the atomic position (RMSD) value between the prepared 3CL^pro^, PL^pro^, and raw 3CL^pro^ and PL^pro^ to determine any significant differences in the structures.

### 5.3. Preparation of Ligands

**Figure 5:**
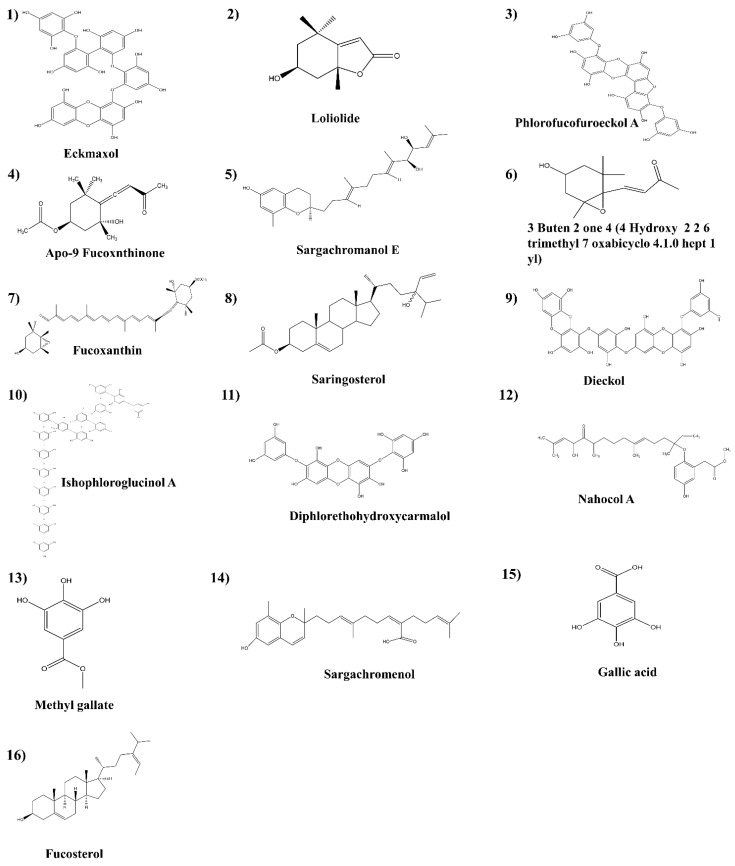
Structures of the ligands.

The 3D structure of each compound was generated and the hydrogen atoms were added. The energy of the ligand was minimized using the “Clean geometry” tool and by applying the CHARMm force field. The final ligand structure generated using the “Prepare ligand” tool was optimized using the DS ligand optimization tool, and the energy of the ligands was minimized using the DS minimization tool. The summary of the ligands presented in this study is shown in Figure 5. (**1.** Eckmaxol, **2.** Loliolide, **3.** Phlorofucofuroeckol A, **4.** Apo-9 Fucoxnthinone, **5.** Sargachromanol E, **6.** 3 Buten 2 one 4 (4 Hydroxy 2 2 6 trimethyl 7 oxabicyclo 4.1.0 hept 1 yl), **7.** Fucoxanthin, **8.** Saringosterol, **9.** Dieckol, **10.** Ishophloroglucinol A, **11.** Diphlorethohydroxycarmalol. **12.** Nahocol A, **13.** Methyl gallate, **14.** Sargachromenol, **15.** Gallic acid, **16.** Fucosterol). The ligands that were selected for validation were 16 compounds obtained from marine algae.

### 5.4. Molecular Docking Analysis

The docking of the selected ligands with prepared proteins was performed using DS. The crystal structure of 3CL^pro^ bound to the N3 inhibitor and PL^pro^ bound to GRl0617 were reproduced, and the RMSD values of the raw crystal structure and docking results were calculated to confirm the accuracy of the process. Initially, flexible docking experiments were performed using the 3D crystal structures of 3CL^pro^ and PL^pro^. Flexible docking is a fully automated workflow process. The flexible docking protocol permits receptor flexibility during the docking of flexible ligands. The side chains of specified amino acids in the target receptor protein are permitted to move during the docking process. Moreover, the receptor was adapted to different ligands in an induced-fit model. Therefore, flexible docking was performed to determine the suitable orientation of the ligand in the active site of each receptor protein. The results obtained from the flexible docking process were used to calculate the binding energy between each ligand and receptor protein using CHARMm-based energy. The free energy of the complex, ligand, and receptor was used to calculate the free energy of the binding.

Energy binding = Energy Complex − (Energy Ligand + Energy Receptor).

The best four ligands were selected for the biological assays based on the results of the molecular docking process.

### 5.5. Sample Collection and Extraction

Brown algae *Ishige okamurae* (IO) and *Ecklonia cava* (EC) were collected from the coastal area of Seongsan, Jeju, South Korea in February 2019. *Ecklonia maxima* (EM) was collected from the coastal area of Cape Town, South Africa in 2019 February. The samples were washed 4 times, immediately after being harvested, with running water to remove any salt, attached sand, and epiphytes. The washed seaweeds were stored at temperatures below −70 °C. The frozen seaweeds were lyophilized using a freeze dryer and the dried seaweeds were ground into powder. Sample extraction was performed using 70% ethanol 3 times at room temperature. The subsequent solution was evaporated using a rotary evaporator and the resulting ethanol extract powders of IO (IOE), EC (ECE), and EM (EME) were obtained. IOE, ECE, and EME were dissolved in deionized water and successfully fractionated using n-hexane, chloroform, ethyl acetate, and butanol, respectively. Each resulting fraction was evaporated and the ethyl acetate fractions of IO (IOEA), EC (ECEA), and EM (EMEA) were utilized to isolate the desired compounds. The centrifugal partition chromatography (CPC 240, Tokyo, Japan) and ODS cartridge in the FlashPrep system (C-850 FlashPrep, BUCHI, Switzerland) were utilized to further separate the IOEA, ECEA, and EMEA.

### 5.6. Isolation of Ishophloroglucin A (IPA) and Diphlorethohydroxycarmalol (DPHC)

Centrifugal partition chromatography (CPC 240, Tokyo, Japan) was utilized to isolate the IPA. The rotor volume was 1 L. The method was continued in a two-phase solvent system, which consisted of n-hexane:ethyl acetate:methanol:water at a 1:9:4.5:6.5 *v/v* ratio. These solvents were thoroughly mixed and equilibrated in a separatory funnel. The upper organic phase acted as a stationary phase and the lower aqueous phase acted as a mobile phase. The CPC instrument was conditioned until it attained hydrostatic equilibrium, and 500 mg of IOEA was dissolved in 6 mL of a 1:1 *v/v* water:methanol ration of the CPC solvent system and injected using an isocratic pump (Kromaton). The effluent was monitored at 230 nm and the fractions were collected into test tubes using a fraction collector (3 min for each tube). All the fractions collected from the same compounds were pooled to continue further purification processes. The high-performance liquid chromatography (HPLC) system (Milford, Massachusetts, USA) equipped with a PDA detector was used for further purification. The semi-preparative column HPLC column YMC-Pack ODS-A 10 mm × 250 mm, 5 µm (YMC Co., Ltd., Kyoto, Japan) was used in an isocratic solvent mode (32% acetonitrile with 1% formic acid), and the flow rate was 2 mL/min [33,34] (Supplementary Figure S11)

### 5.7. Isolation of Dieckol

Dieckol was isolated from ECEA using an ODS cartridge in the Flashprep system (C-850 FlashPrep, BUCHI, Switzerland) equipped with PDA and ELSD detectors. A packing PREP C18, 55–105 µm, 125 Å (Waters, Milford, CT, USA) column was used with a 20 mL/min flow rate. The mobile phase consisted of water and acetonitrile with a gradient method (0 min 90:10 *v/v*, 0–12 min 90:10 *v/v*, 12–36 min 85:15 *v/v*, 36–68 min 80:20 *v/v*, 68–80 min 0:100 *v/v*). The fractions were collected based on the results obtained at 230 nm (Supplementary Figure S12).

### 5.8. Isolation of Eckmaxol

Eckmaxol was isolated from EMEA using the centrifugal partition chromatography (CPC 240, Tokyo, Japan) method composed of n-hexane:ethyl acetate:methanol:water at a ratio of 3:7:4:6: *v/v*. The abovementioned solvents were vigorously mixed and equilibrated to separate two phases at room temperature. The upper organic phase was used as a stationary phase and the lower aqueous phase was used as a mobile phase. The organic stationary phase was filled into the CPC column and rotated at 1000 rpm, and the aqueous mobile phase was pumped into the column in a descending mode at a flow rate of 2 mL/min. The hydrodynamic equilibrium was maintained before injecting the sample, and 500 mg of EMEA dissolved in 6 mL of a 1:1 *v/v* ratio of water:methanol was injected through the injection valve. The automatic fraction collector was utilized to collect the fractions (6 mL for each tube) under the 230 nm UV detection range. The HPLC system equipped with a PDA detector was used for further purification processes. A YMC-Pack ODS-A 10 mm × 250 mm, 5 µm column with acetonitrile +0.1% formic acid and deionized water + 0.1% formic acid was used as a mobile phase, and the flow rate was 2 mL/min [35] (Supplementary Figure S13).

### 5.9. In Vitro Cleavage Inhibition Assay

The inhibitory activity of the compounds of interest was measured using the “3CL^pro^ and PL^pro^ (SARS-CoV-2)” assay kit (AMSBIO, Madrid, Spain). The compounds were dissolved in dimethyl sulfoxide (DMSO) and diluted into the assay buffer available with the kit. The final DMSO concentration of the highest concentration of each compound used in the assay was lower than 1%. IPA, Dieckol, and Eckmaxol were incubated with 120 ng of the 3CL^pro^ enzyme for 30 min at room temperature with slow shaking. Broad-spectrum antiviral medication GC376 was used as a test inhibitor in the assay, and 50 µM of the fluorogenic substrate was added to each well to determine the 3CL^pro^ inhibitory activity. The diluted concentrations of IPA, Dieckol, and Eckmaxol were treated into each well that contained 1.2 ng of PL^pro^ enzyme and incubated for 30 min with slow shaking at room temperature. The blank well was treated with only 25 µM of fluorogenic substrate and the positive control well contained only PL^pro^ enzyme and fluorogenic substrate to evaluate the PL^pro^ inhibitory activity. The experiment was performed on a 96-well plate. The negative control was the well that only contained 3CL^pro^ or PL^pro^ enzymes, and the fluorogenic substrate used to measure the enzyme activity and blank well contained only substrate. The enhanced fluorescence emission as a result of substrate cleavage was monitored at an excitation of 360 nm and emission of 460 nm using a Synergy HTX multi-mode microplate reader (Winooski, VT, USA).

The IC_50_ value of each compound was calculated and the experimental data were fit to a logistic curve using the equation below:

Enzyme activity % = [S − B]/[P − B] × 100%

“B” is the fluorescence of the blank (substrate and assay buffer), “P” is the fluorescence of the negative control (substrate and enzyme), and “S” is the fluorescence of the tested sample.

### 5.10. Enzyme Kinetic Evaluation

The enzyme kinetic mechanism of these isolated compounds was studied with a series of substrate concentrations with various concentrations of inhibitors. The data were plotted in the graph (*y* axis—1/V and *x* axis—1/[S]).

### 5.11. Cell-Based Inhibition Assay

The enzyme kinetic mechanism of the interaction between the isolated compounds and 3CL^pro^ and PL^pro^ was evaluated with various inhibitor concentrations and substrate concentration series.

#### 5.11.1. Cell Culture

Vero-E6 cells were purchased from the American Type Culture Collection (ATCC, Manassas, VA, USA). Cells were cultured using Dulbecco’s modified Eagle’s medium (DMEM) (Gibco/BRL; Burlington, ON, Canada) containing 10% FBS and 1% antibiotics under 37 ºC and 5% CO_2_.

#### 5.11.2. 3CL^pro^ and PL^pro^ Cell-Based Cleavage Inhibition Assay

The 3CL^pro^ and PL^pro^ genes were fused in-frame with a cleavage site and a firefly luciferase gene at the C-terminus. Both genes were purchased from SinoBiological (Taizhou, Zhejiang, China). The 3CL^pro^ gene was amplified using PCR with designed forward and reverse primers 5′-GAGAGAGCGGCCGCATGGCATTCCCATCTGGTAAAGTTGAGG- 3′ and 5′-GAGAGAGGATCCCCTTCCTGAAGCCGCTCTGCAGCACGGCGCTTTGGAAAGTAACACCTGAGCATTGTCTAACAAC-3′, respectively. The PL^pro^ gene was amplified using PCR with designed forward and reverse primers 5′-GAGAGAGCGGCCGCATGGAAGTGAGGACTATTAAGGTGTTTACAACAG-3′ and 5′-GAGAGAGGATCCCGCACATGGCGCCGCCCCTCAGCCTAACTGGTTTTATGGTTGTTGTGTAACTGTTTTCTTTG, respectively.

**Figure 6:**
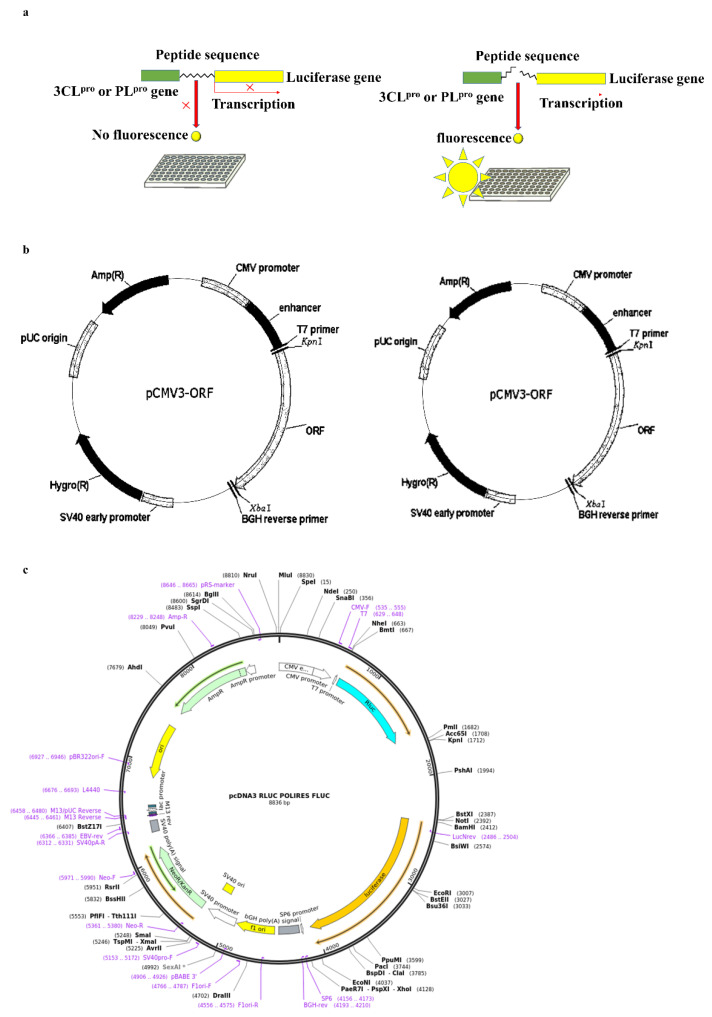
Cell-based 3CLpro- and PLpro-cleavage inhibition assay. (**a**) Principle of the assay, (**b**) plasmid containing 3CLpro and PLpro genes, and (**c**) vector containing firefly and renilla luciferases.

The forward primer consisted of a NotI restriction site and the reverse primer contained a BamHI restriction site and in-frame gene encoding for the 3CL^pro^-cleavage site (AGCGCCGTGCTGCAGAGCGGCTTCAGGAAG) luciferase gene and PL^pro^-cleavage site (AGGCTGAGGGGCGGCGCCATGTGC) luciferase gene. The plasmid that contained the 3CL^pro^ gene was amplified by PCR using the abovementioned primers and cloned into the NotI/BamHI restriction sites of the pcDNA RLUC POLIRES FLUC vector (addgene, Watertown, MA, USA). The recombinant plasmid was transfected into Vero-E6 cells using an X-tremeGENE HP DNA transfection reagent (Sigma-Aldrich, St. Louis, MO, USA). Vero-E6 cells at 80–90% confluence in 24-well plates were transfected with 0.25 µg of total plasmid per 1 well that contained DMEM with 0% FBS and 0% antibiotics. The media was replaced with DMEM that contained 10% FBS and 1% antibiotics following 23 h of incubation (37 °C and 5% CO_2_) and the samples were treated. The dual-luciferase reporter assay system (Promega) was utilized to evaluate the expression of firefly luciferase activity after an 18–20 h incubation (37 °C and 5% CO_2_) period. The expression level of firefly and renilla luciferases was measured by a Luminometer (Figure 6).

### 5.12. Cytopathic Effect (CPE) Reduction Evaluation

The CPE reduction assay was performed according to the previously reported method [36]. Briefly, the cells were seeded with 5 × 10^4^ cells/well in a 96-well plate and incubated at 37 °C for 48 h. A total of 100 µL of the virus was treated in each well using DMEM media that was FBS free and 1% antibiotic to infect a 0.01 multiplicity of infection (MOI) and incubated for an additional 1 h at 37 °C. Subsequently, the infected cells were treated with samples after removing the virus and 72 h of incubation was performed, and the MTT solution was added and allowed to stand for a 4 h incubation period. The formazan crystals were dissolved and the absorbance was measured. The cytotoxicity ratio was calculated according to the following equation:

Virus inhibition rate (%) = ((Test OD − Virus OD)/(Control OD − Virus OD)) × 100%

### 5.13. Statistical Analysis

All the compounds were examined in the set of triplicate experiments. IC_50_ (50% inhibitory concentration) values of the compounds represent the concentration that caused 50% enzyme activity loss. Using a minimum of three samples, standard deviation was calculated in all the experiments. The inhibition mechanism of the compounds was determined by comparing the statistical results, including the Akaike’s information criterion values, of different inhibition models and selecting the one with the best fit [37].

## Figures and Tables

**Table 1:** The cDocker interaction energies and free binding energies (kcal/mol) of selected ligands obtained from marine algae with the 3CLpro receptor protein.

No.	Sample Name	cDocker Interaction Energy (kcal/mol)	Binding Energy (kcal/mol)
1	GC376	−58.7189	−182.685
2	N3 inhibitor	−72.5369	−185.054
3	Ishophloroglucinol A	−63.1128	−186.875
4	Diphlorethohydroxycarmalol	−63.1128	−158.462
5	Dieckol	−68.0895	−257.388
6	Phlorofucofureckol-A	-	-
7	Nahocol A	−48.4205	−119.254
8	Saringosterol	−51.1228	−100.977
9	Sargacromanol E	−54.4594	−118.902
10	Fucoxanthin	-	-
11	Eckmaxol	−7.9218	−235.86
12	Fucosterol	−51.7309	−93.9243
13	Gallic acid	−28.1739	−141.556
14	Methyl gallate	−39.1007	−98.995
15	Apo-9 fucoxanthinone	-	-
16	3-Buten-2-one,4-(4-hydroxy-2,2,6-trimethyl-7-oxabicyclo [4.1.0]hept-1-yl)-	−27.7698	−62.684
17	Loliolide	−21.163	−93.869
18	Sargachromenol	−64.3135	−91.963

**Table 2:** The cDocker interaction energies and free binding energies (kcal/mol) of selected ligands obtained from marine algae with the PLpro receptor protein.

No.	Sample Name	cDocker Interaction Energy (kcal/mol)	Binding Energy (kcal/mol)
1	GRL0617	−74.2579	−133.288
2	Ishophloroglucinol A	−43.5452	−271.055
3	Diphlorethohydroxycarmalol	−114.898	−146.253
4	Dieckol	−65.5972	−191.131
5	Phlorofucofuroeckol-A	−74.2645	−110.355
6	Nahocol A	−44.5236	−110.496
7	Saringosterol	−41.3209	−77.7694
8	Sargacromanol E	−54.1180	−15.2530
9	Fucoxanthin	-	-
10	Eckmaxol	−72.3064	−169.8830
11	Fucosterol	−34.7463	−71.5172
12	Gallic acid	−31.6026	−63.3519
13	Methyl gallate	−35.2097	−35.6745
14	Apo-9 fucoxanthinone	-	-
15	3-Buten-2-one,4-(4-hydroxy-2,2,6-trimethyl-7-oxabicyclo [4.1.0]hept-1-yl)-	−33.9821	−74.9392
16	Loliolide	−21.1630	−93.8690
17	Sargachromenol	−47.2003	−115.477

**Table 3:** Inhibitory activity of isolated compounds on the cell-free cleavage of 3CL^pro^ and PL^pro^.

No.	Drug Target	Ligand	IC_50_ Value (µM)
1	3CL^pro^	Ishophloroglucin A	0.4814 ± 0.031
2		Dieckol	5.4902 ± 0.092
3		Eckmaxol	1.8886 ± 0.078
4		Diphlorethohydroxycarmalol	3.1193 ± 0.066
5		GC376	0.4231 ± 0.045
6	PL^pro^	Ishophloroglucin A	1.4048 ± 0.007
7		Dieckol	19.7404 ± 0.090
8		Eckmaxol	19.8349 ± 0.121
9		Diphlorethohydroxycarmalol	6.6367 ± 0.056
10		GRL0617	1.5 ± 0.120

## Data Availability

Not applicable.

## Supplementary Materials

The following supporting information can be downloaded at: https://www.mdpi.com/article/10.3390/md20120786/s1, Supplementary Figure S1: In-silico analysis of 3CL^pro^ with N3 inhibitor, Supplementary Figure S2: In-silico analysis of PL^pro^ with GRL0617 inhibitor, Supplementary Figure S3: In-silico analysis of 3CL^pro^ with Ishophloroglucinol A (IPA), Supplementary Figure S4: In-silico analysis of 3CL^pro^ with Dieckol, Supplementary Figure S5: In-silico analysis of 3CL^pro^ with Eckmaxol, Supplementary Figure S6: In-silico analysis of 3CL^pro^ with Diphlorethohydroxycarmalol (DPHC), Supplementary Figure S7: In-silico analysis of PL^pro^ with Ishophloroglucinol A (IPA), Supplementary Figure S8: In-silico analysis of PL^pro^ with Diekcol, Supplementary Figure S9: In-silico analysis of PL^pro^ with Eckmaxol, Supplementary Figure S10: In-silico analysis of PL^pro^ with Diphlorethohydroxycarmal (DPHC), Supplementary Figure S11: Extaction and isolation procedure of Ishophloroglucinol A (IPA) and Diphlorethohydroxycarmalol (DPHC) from *Ishige okamurae,* Supplementary Figure S12: Extraction and isolation procedure of Dieckol from *Ecklonia cava,* Supplementary Figure S13: Extraction and isolation procedure of Eckmaxol from *Ecklonia maxima***.**
