# Supplementary material for: Polyphenolic Compounds Isolated from Marine Algae Attenuate the Replication of SARS-CoV-2 in the Host Cell through a Multi-Target Approach of 3CLpro and PLpro
Source: Mar Drugs. 2022 Dec 19;20(12):786. doi: 10.3390/md20120786 (PMC9781010; doi:10.3390/md20120786)
Supplement: Supplementary file 1 [file marinedrugs-20-00786-s001.zip › marinedrugs-2070668-supplementary.pdf]

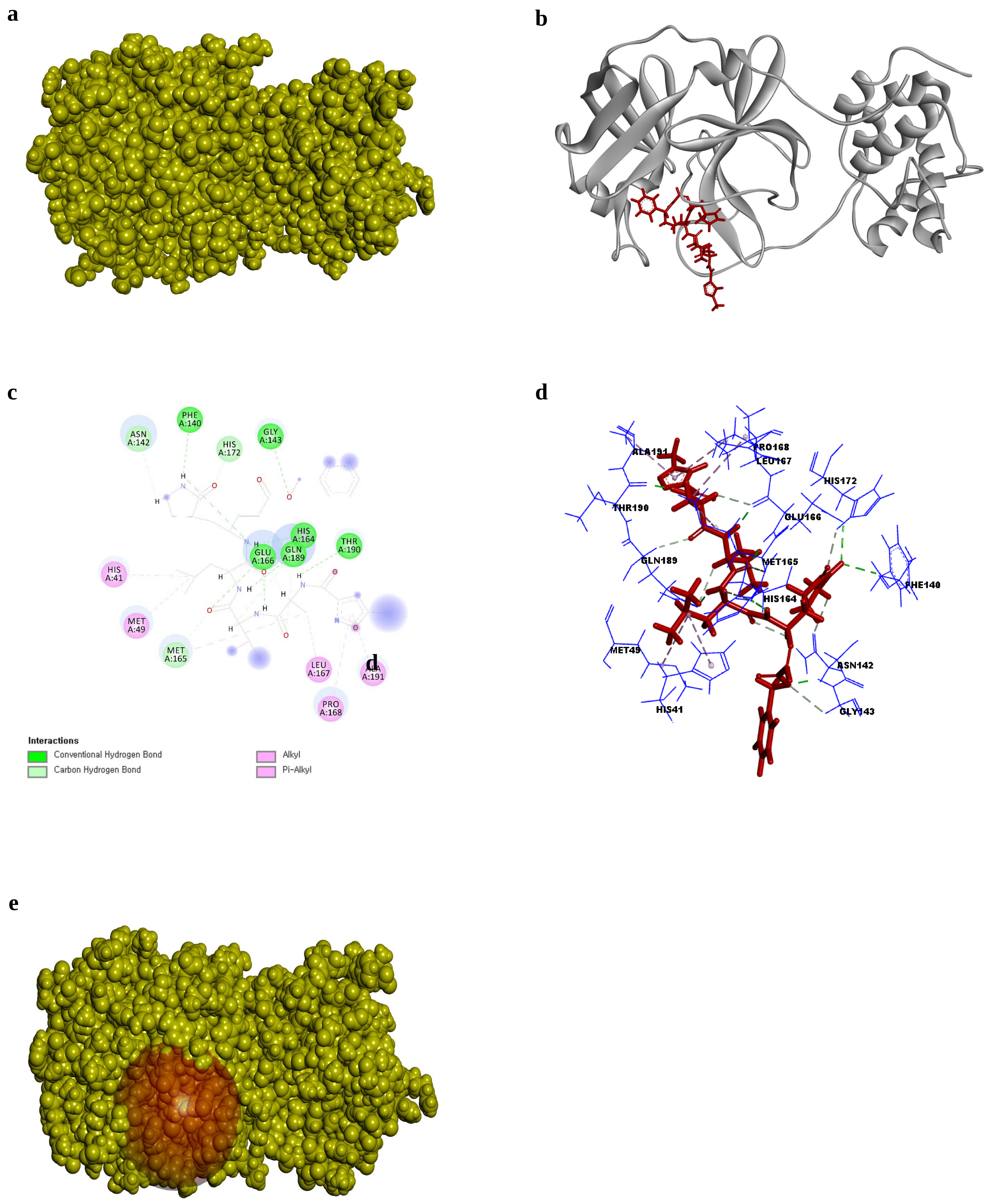

Supplementary Figure S1. *In-silico* analysis of 3CL^{pro} with N3 inhibitor, a) Surface representation 3CL^{pro} and N3 inhibitor ligand complex 3CL^{pro} receptor protein is in yellow color and N3 inhibitor in red color. b) Cartoon representation of 3CL^{pro} and N3 inhibitor ligand complex. 3CL^{pro} receptor protein is in yellow color and N3 inhibitor is in red color. c) 2D representation of ligand interaction between 3CL^{pro} and N3 inhibitor. d) 3D representation of ligand interaction between 3CL^{pro} and N3 inhibitor. e) Prepared active site of 3CL^{pro}.

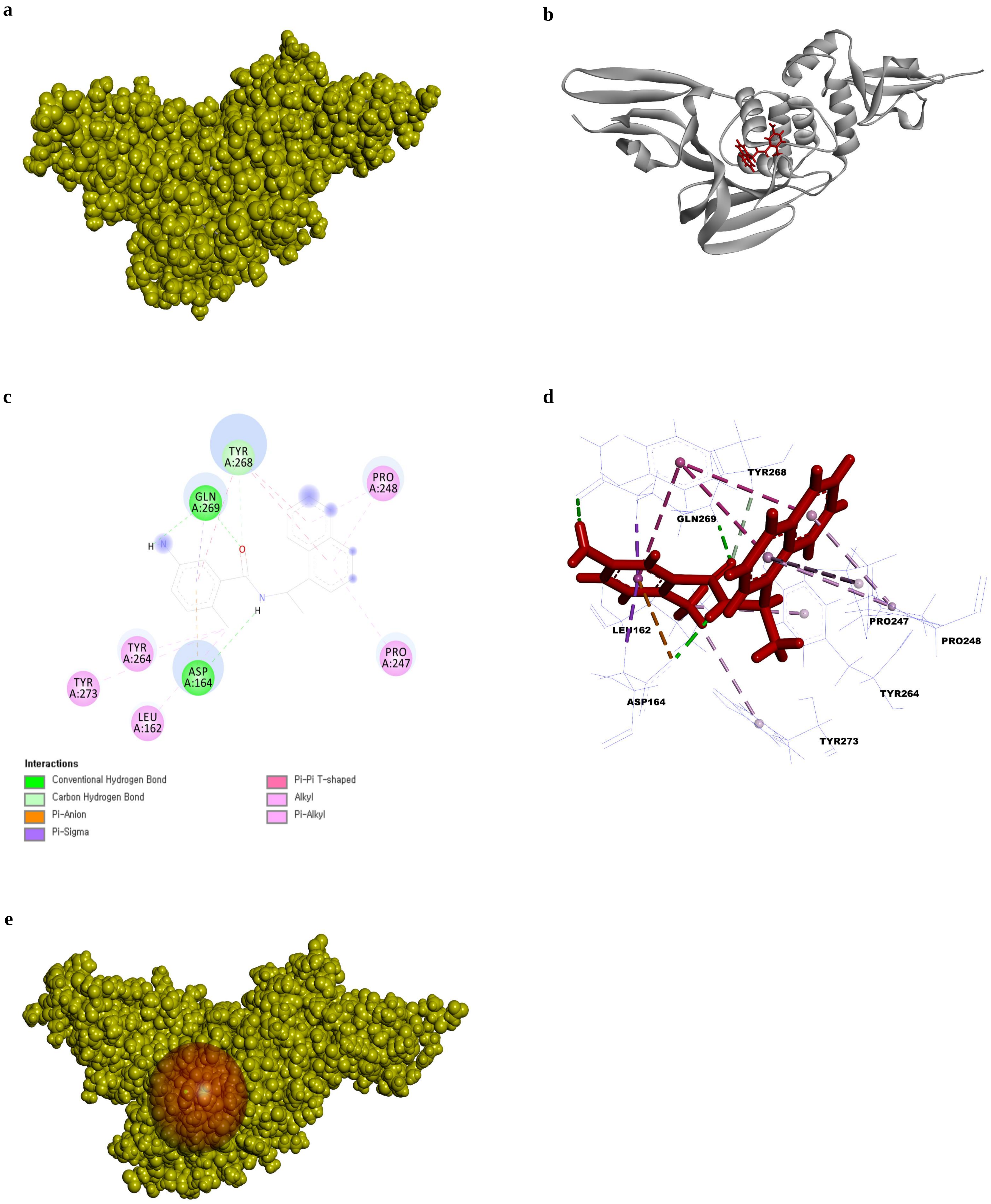

Supplementary Figure S2. *In-silico* analysis of PL^{pro} with GRL0617 inhibitor, a) Surface representation PL^{pro} and GRL0617 inhibitor ligand complex PL^{pro} receptor protein is in yellow color and GRL0617 inhibitor in red color. b) Cartoon representation of PL^{pro} and GRL0617 inhibitor ligand complex. PL^{pro} receptor protein is in yellow color and GRL0617 inhibitor is in red color. c) 2D representation of ligand interaction between PL^{pro} and GRL0617 inhibitor. d) 3D representation of ligand interaction between PL^{pro} and GRL0617 inhibitor. e) Prepared active site of PL^{pro}.

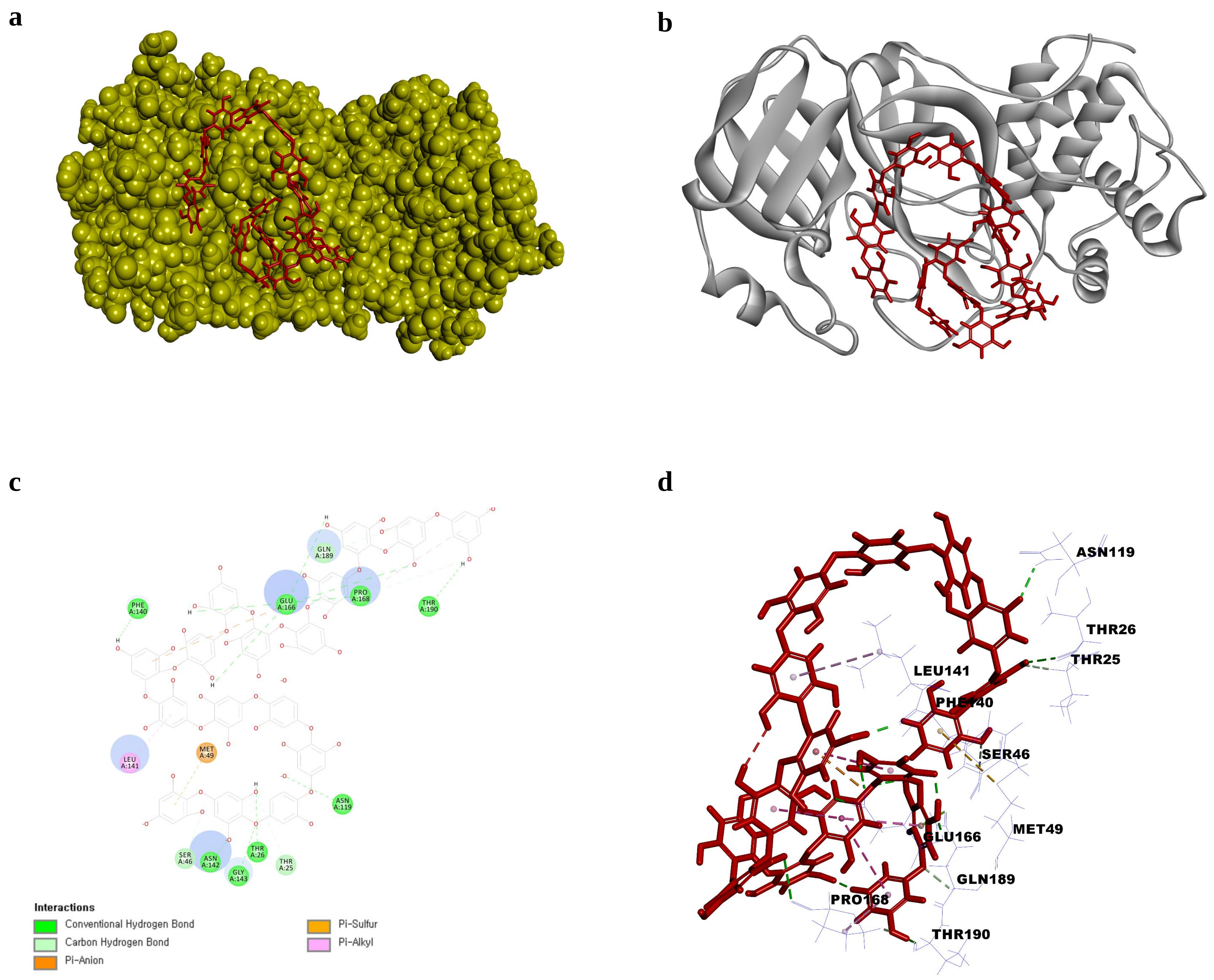

Supplementary Figure S3. ***In-silico*** **analysis of $3CL^{pro}$ with (Ishophloroglucinol A) IPA,** a) 3D representation of docking pose of IPA with $3CL^{pro}$. b) Cartoon representation of docking pose of IPA with $3CL^{pro}$. c) 2D representation of Ligand interaction of IPA with $3CL^{pro}$. d) 3D representation of Ligand interaction of IPA with $3CL^{pro}$

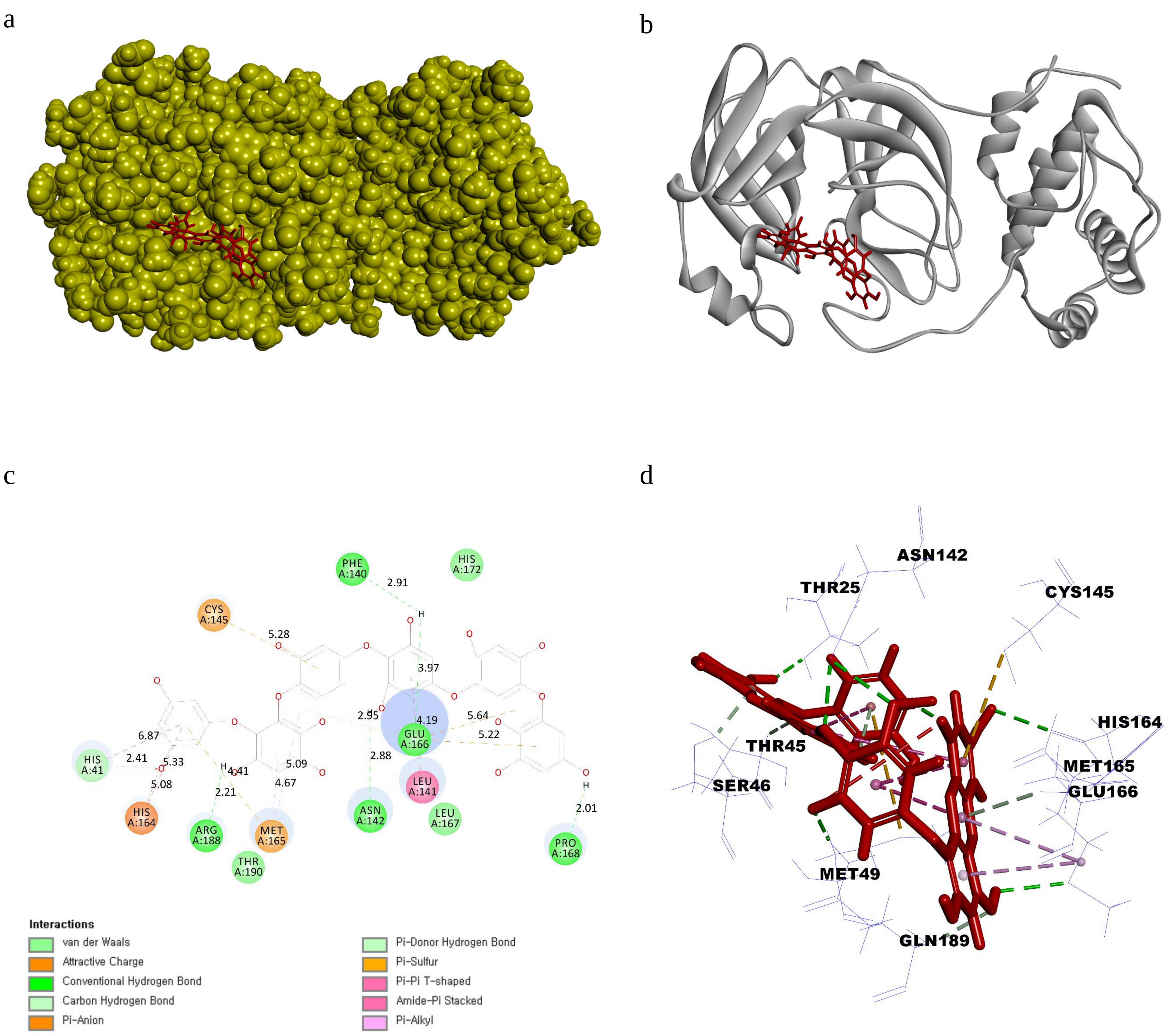

Supplementary Figure S4. *In-silico* analysis of 3CL^{pro} with Dieckol, a) 3D representation of docking pose of Dieckol with 3CL^{pro}. b) Cartoon representation of docking pose of Dieckol with 3CL^{pro}. c) 2D representation of Ligand interaction of Dieckol with 3CL^{pro}. d) 3D representation of Ligand interaction of Dieckol with 3CL^{pro}

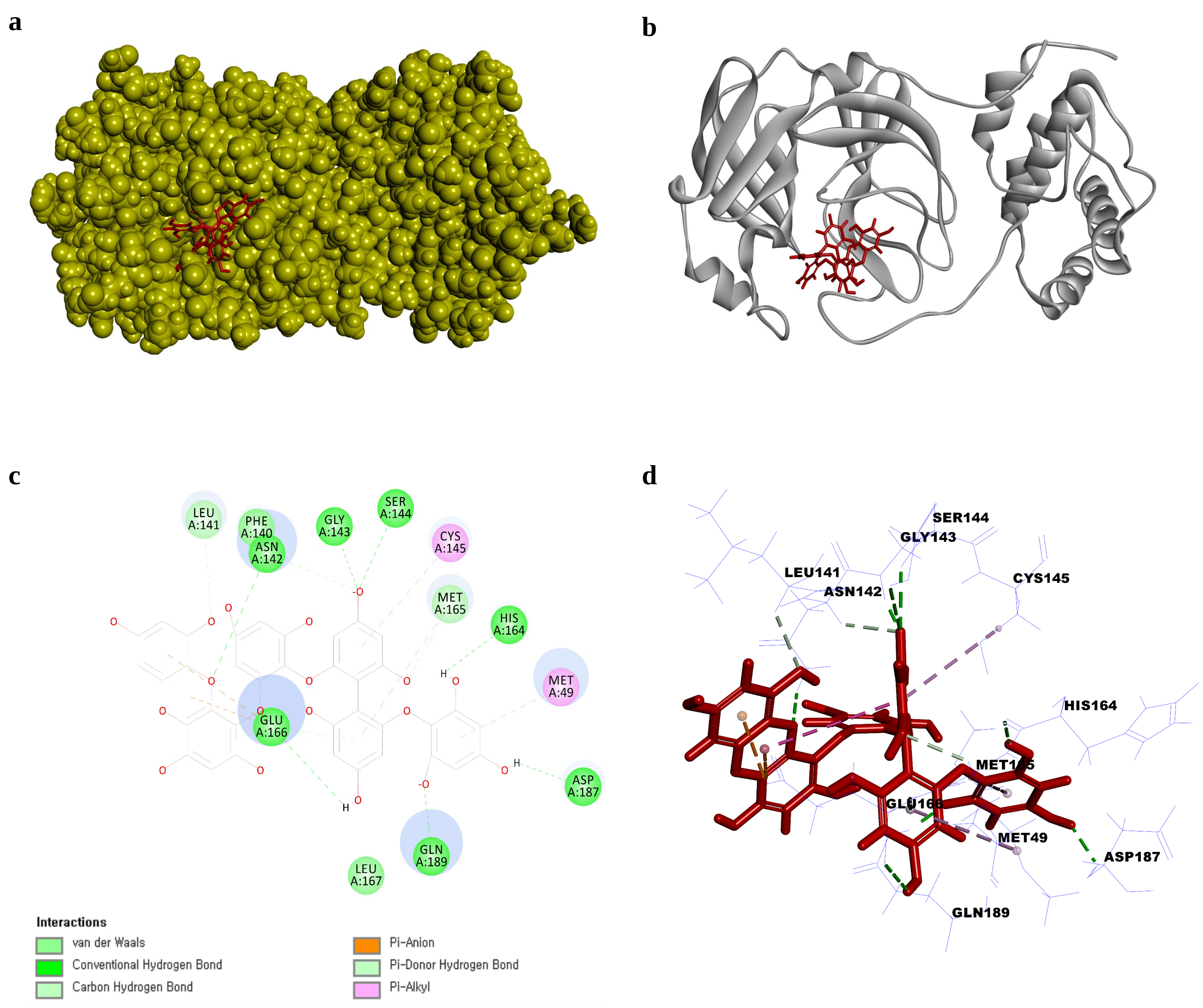

Supplementary Figure S5. ***In-silico*** **analysis of $3CL^{pro}$ with Eckmaxol,** a) 3D representation of docking pose of Eckmaxol with $3CL^{pro}$. b) Cartoon representation of docking pose of Eckmaxol with $3CL^{pro}$. c) 2D representation of Ligand interaction of Eckmaxol with $3CL^{pro}$. d) 3D representation of Ligand interaction of Eckmaxol with $3CL^{pro}$

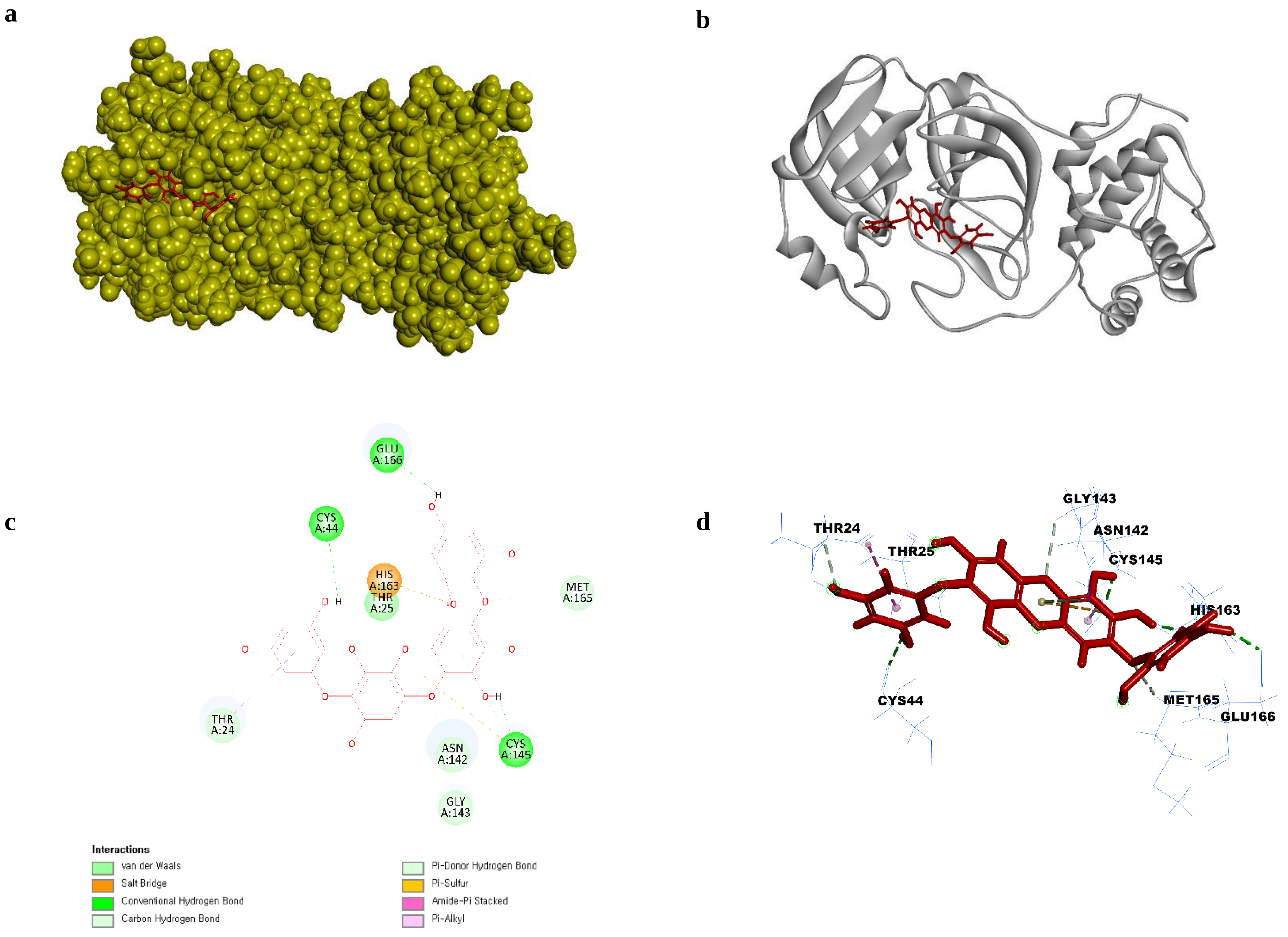

Supplementary Figure S6. ***In-silico*** **analysis of 3CL^{pro} with Diphlorethohydroxycarmalol (DPHC),** a) 3D representation of docking pose of DPHC with 3CL^{pro}. b) Cartoon representation of docking pose of DPHC with 3CL^{pro}. c) 2D representation of Ligand interaction of DPHC with 3CL^{pro}. d) 3D representation of Ligand interaction of DPHC with 3CL^{pro}

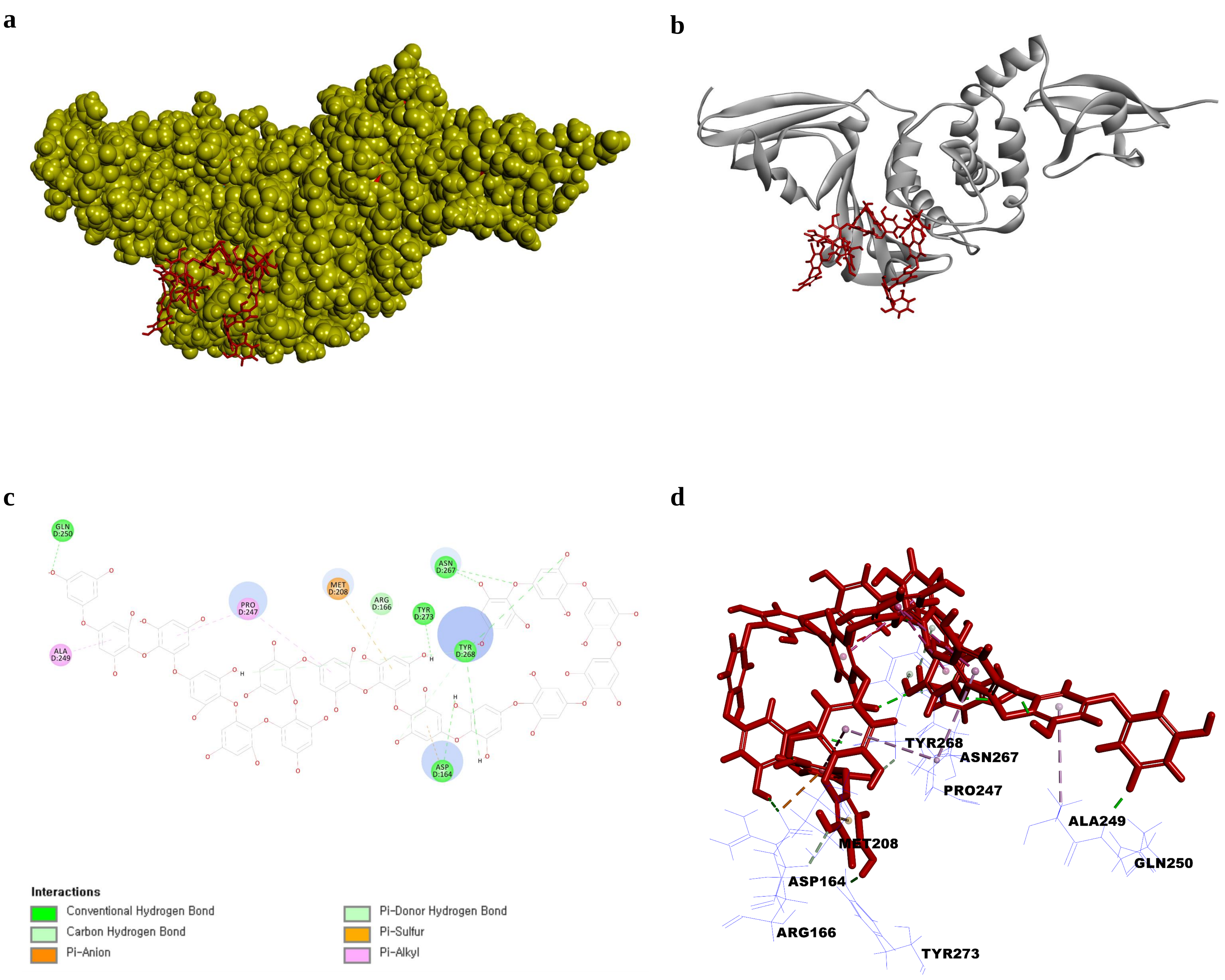

Supplementary Figure S7. *In-silico* analysis of PL^{pro} with Ishophloroglucinol A (IPA), a) 3D representation of docking pose of IPA with PL^{pro}. b) Cartoon representation of docking pose of IPA with PL^{pro}. c) 2D representation of Ligand interaction of IPA with PL^{pro}. d) 3D representation of Ligand interaction of IPA with PL^{pro}

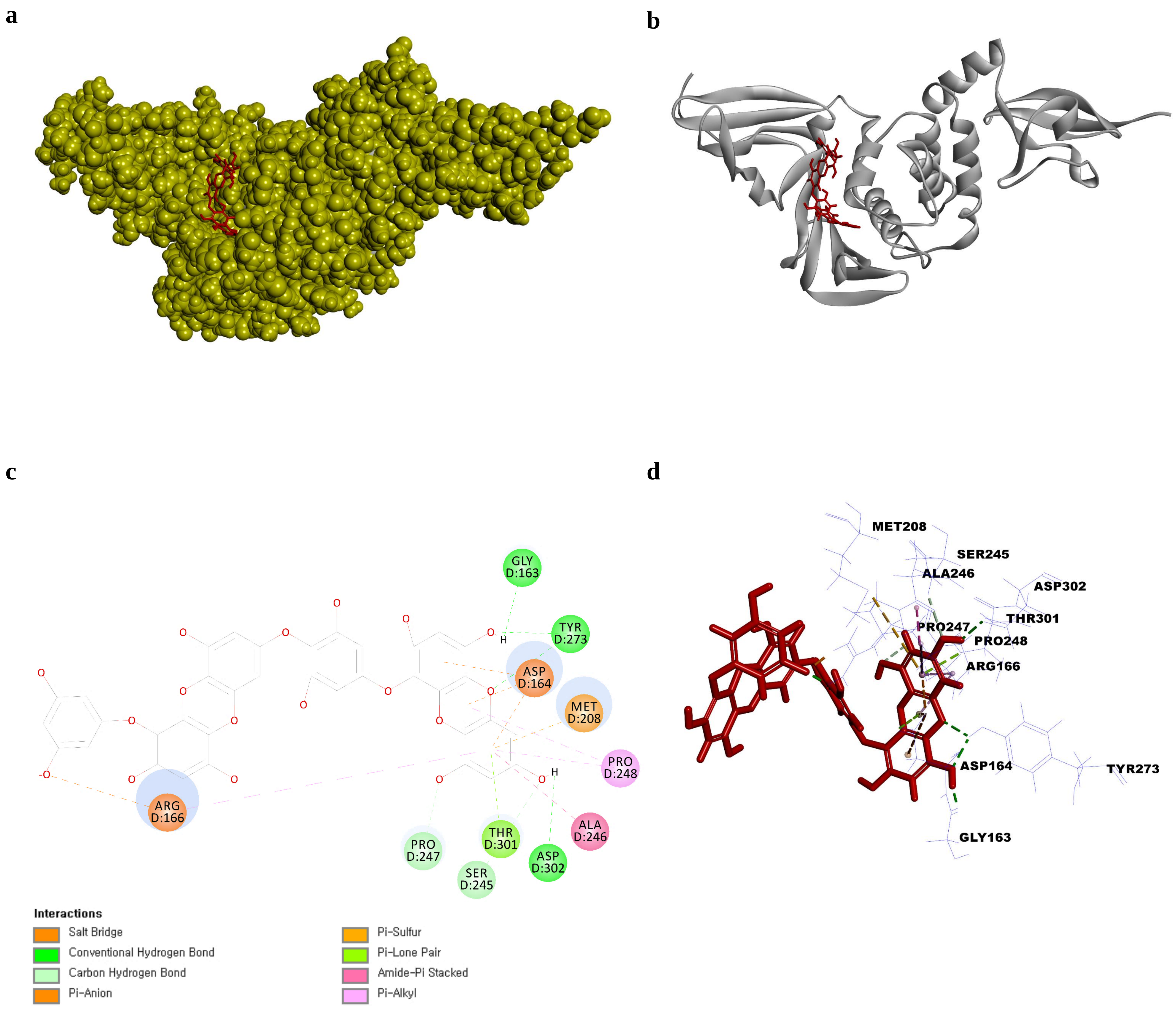

Supplementary Figure S8. *In-silico* analysis of PL^{pro} with Dieckol, a) 3D representation of docking pose of Dieckol with PL^{pro}. b) Cartoon representation of docking pose of Dieckol with PL^{pro}. c) 2D representation of Ligand interaction of Dieckol with PL^{pro}. d) 3D representation of Ligand interaction of Dieckol with PL^{pro}

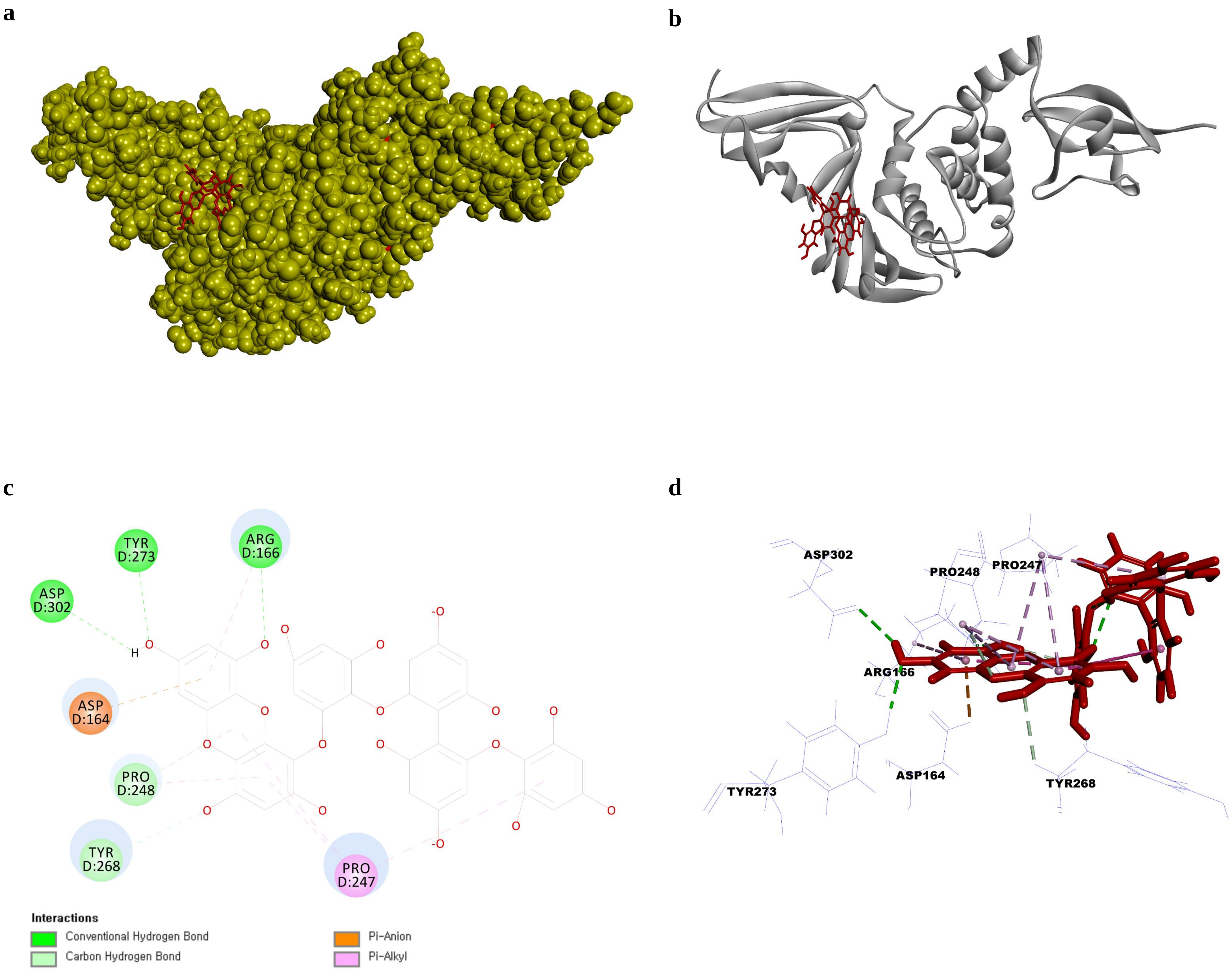

Supplementary Figure S9. *In-silico* analysis of PL^{pro} with Eckamxol, a) 3D representation of docking pose of Eckmaxol with PL^{pro}. b) Cartoon representation of docking pose of Eckmaxol with PL^{pro}. c) 2D representation of Ligand interaction of Eckmaxol with PL^{pro}. d) 3D representation of Ligand interaction of Eckmaxol with PL^{pro}

a

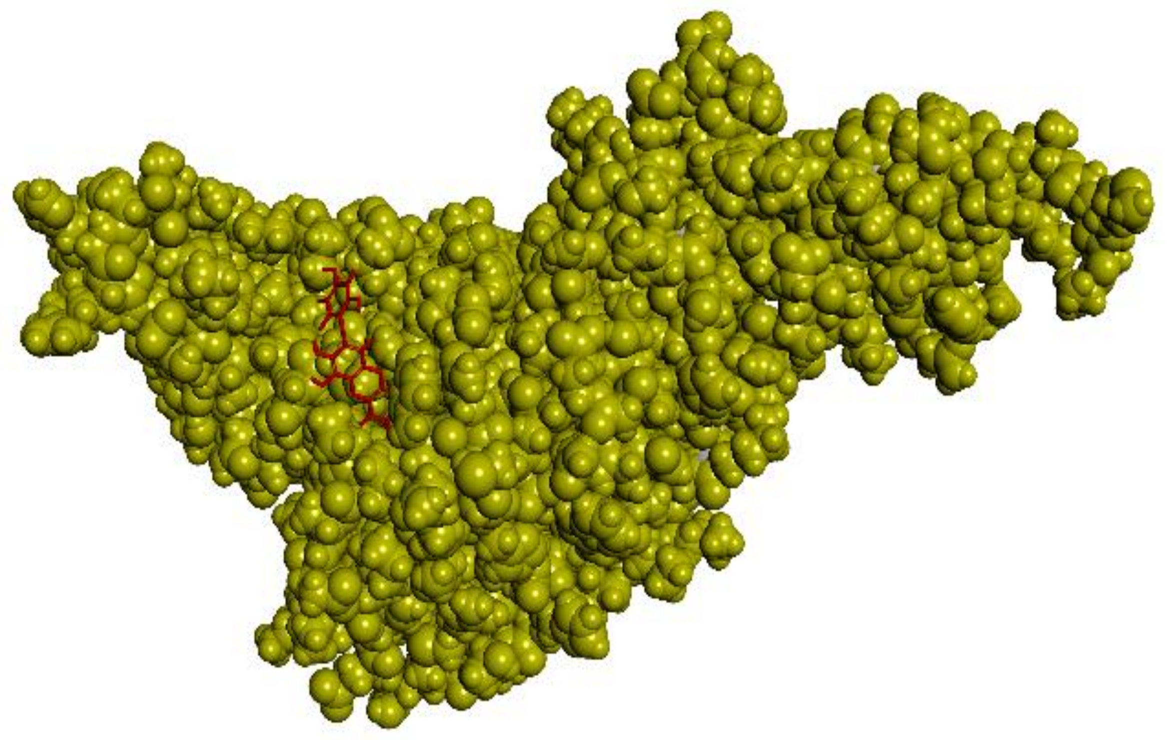

b

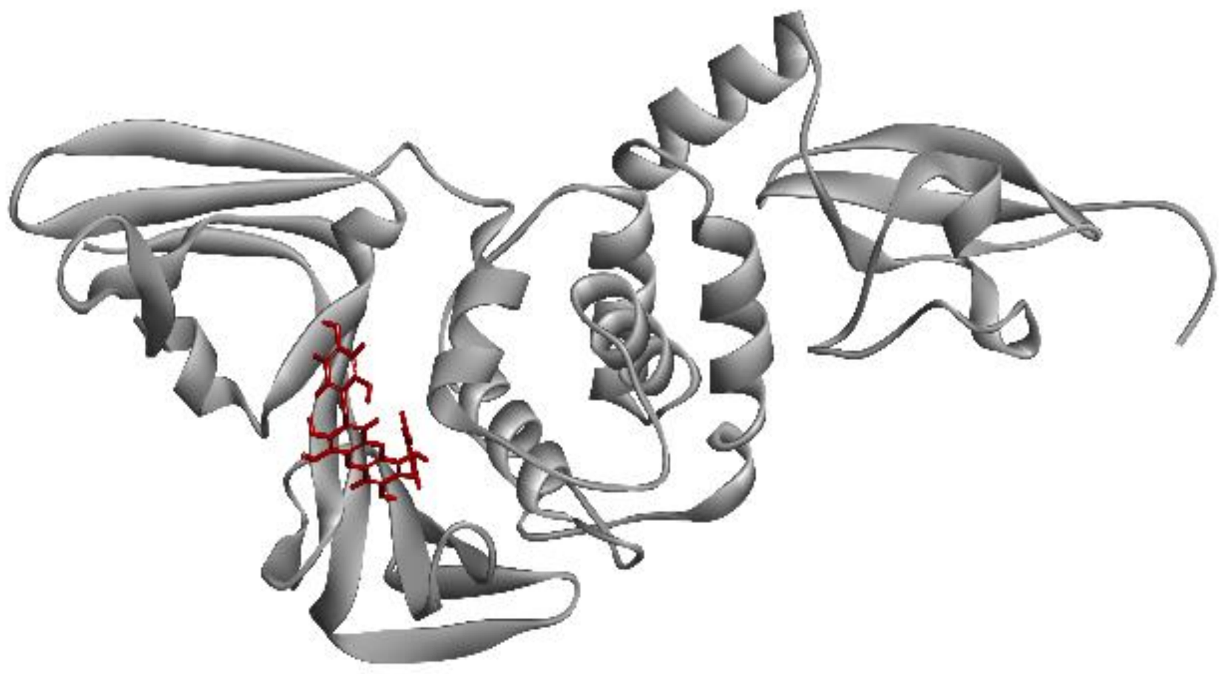

c

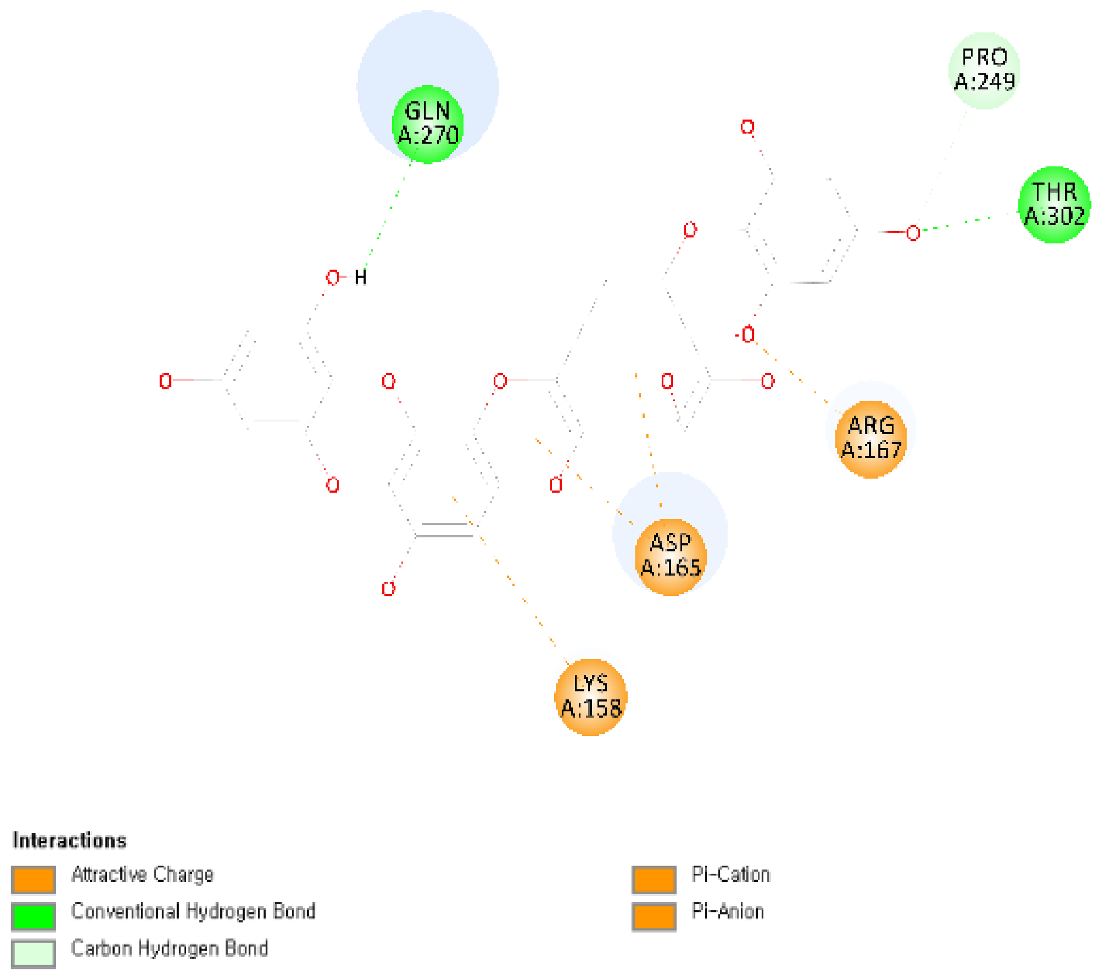

d

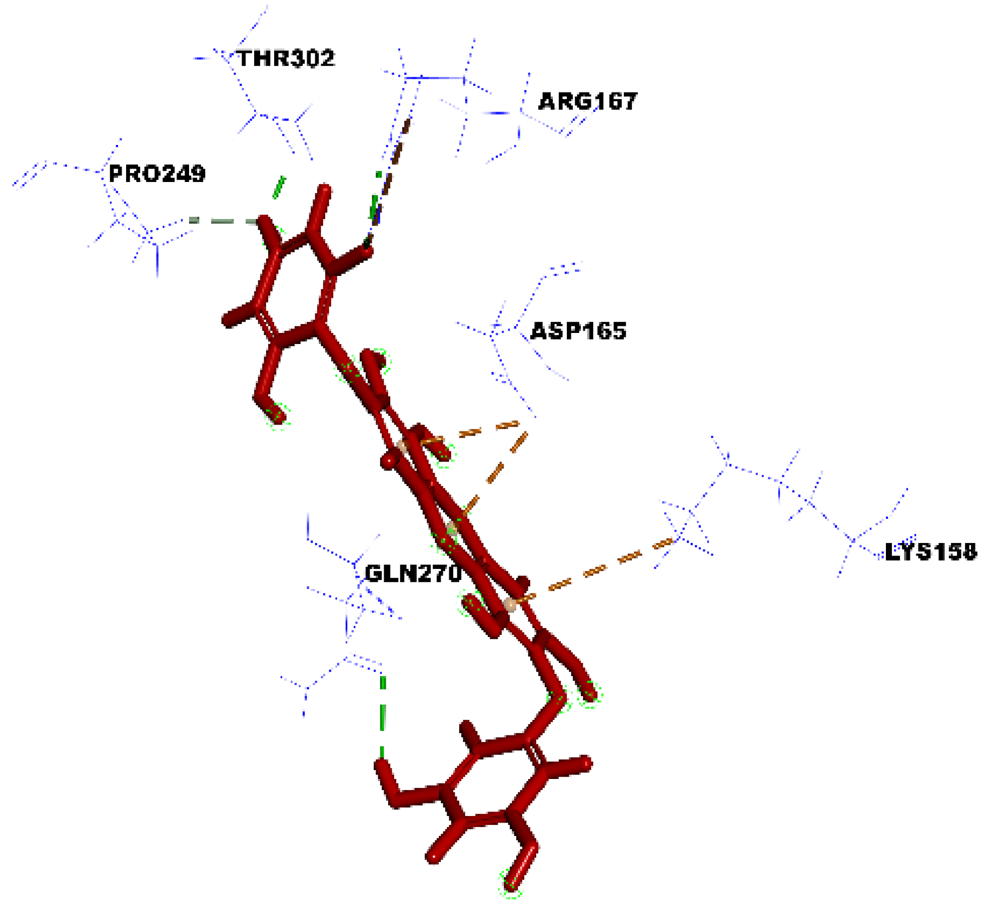

Supplementary Figure S10. *In-silico* analysis of PL^{pro} with Diphlorethohydroxycarmalol (DPHC), a) 3D representation of docking pose of DPHC with PL^{pro}. b) Cartoon representation of docking pose of DPHC with PL^{pro}. c) 2D representation of Ligand interaction of DPHC with PL^{pro}. d) 3D representation of Ligand interaction of DPHC with PL^{pro}

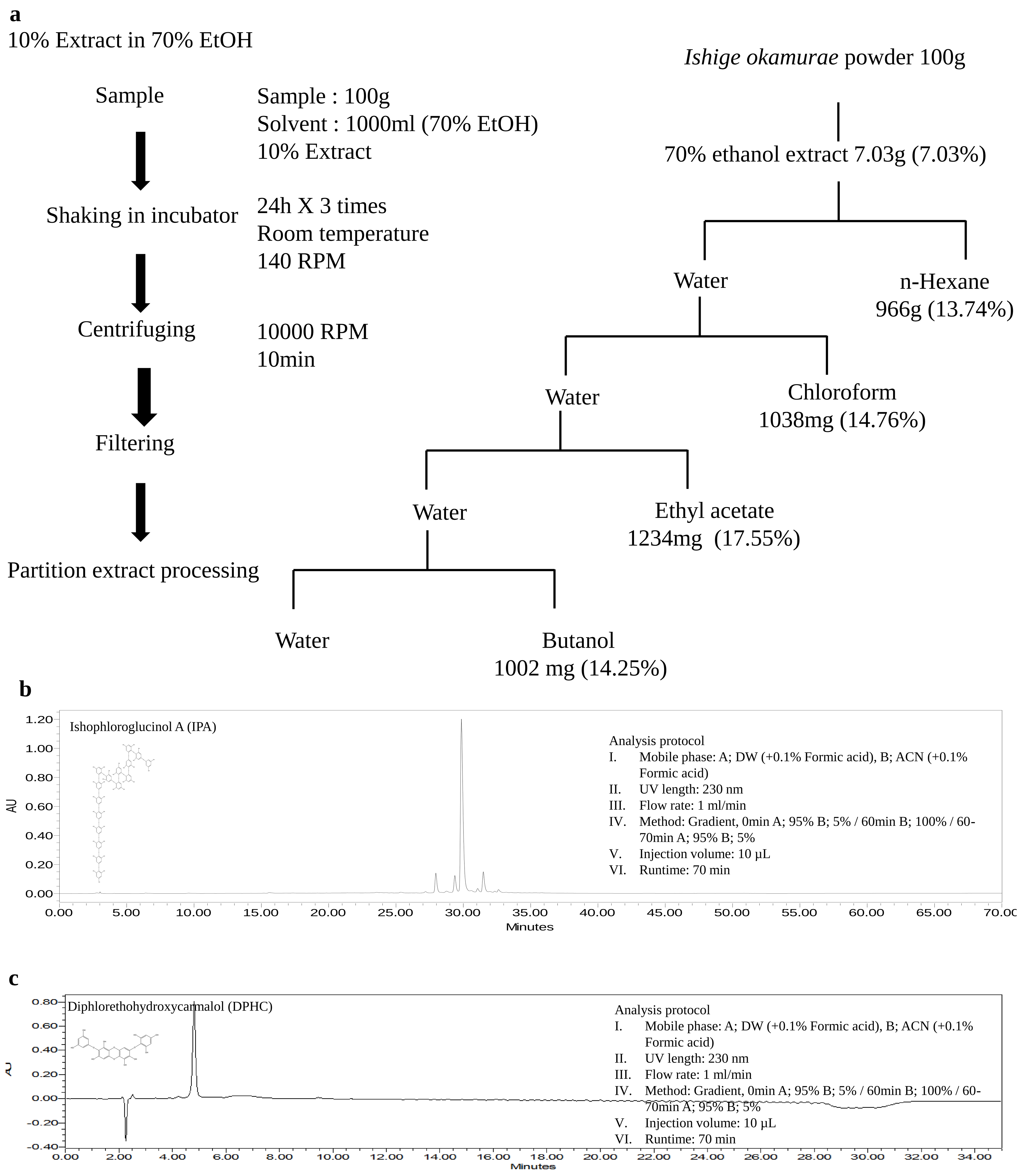

Supplementary Figure S11. Extaction and isolation procedure of Ishophloroglucinol A (IPA) and Diphlorethohydroxycarmalol (DPHC) from *Ishige okamurae*, (a) Extraction protocol, (b) High Performance Liquide Chromatography (HPLC) analysis of IPA, and (c) DPHC.

a

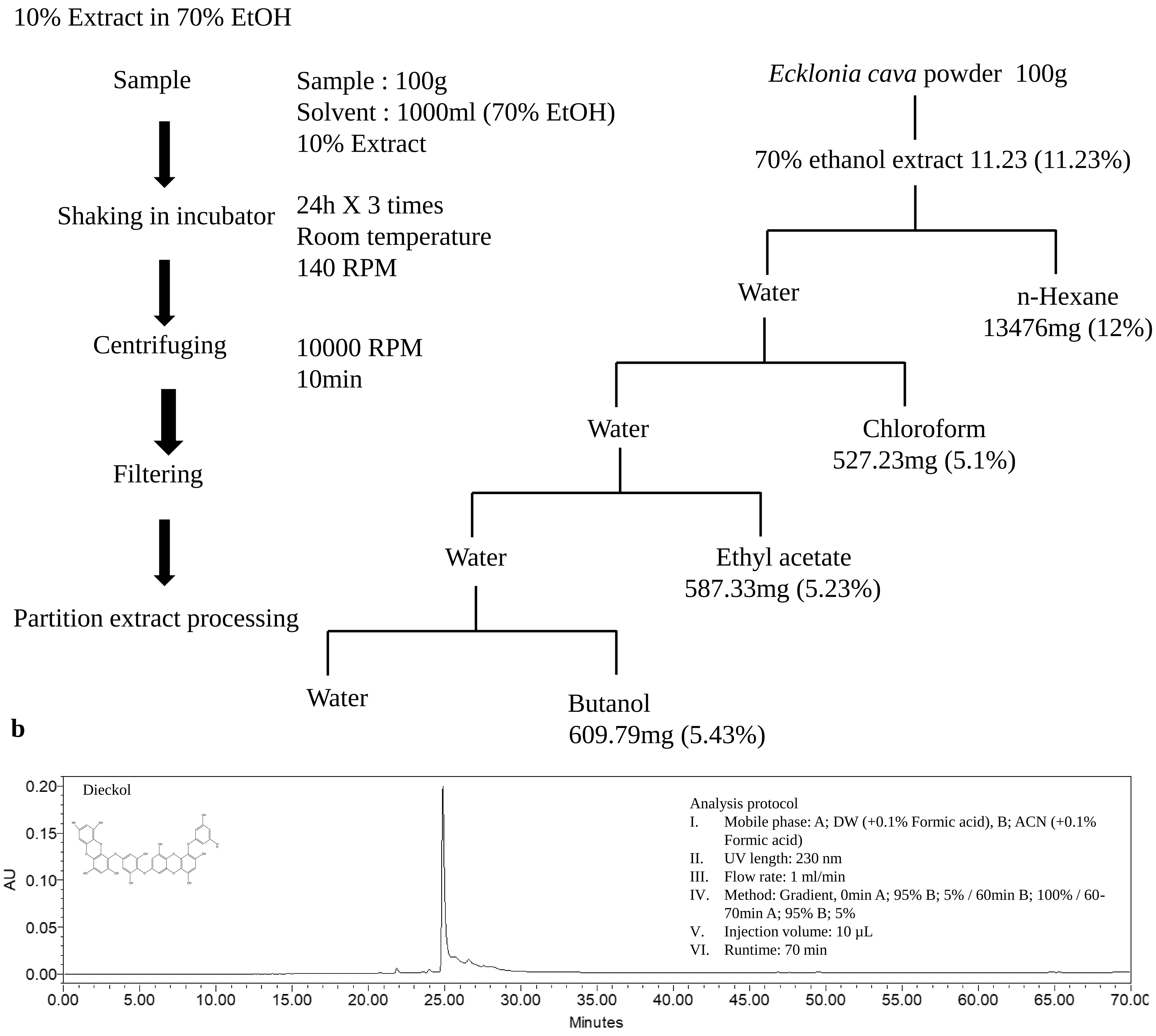

Supplementary Figure S12.. Extaction and isolation procedure of Dieckol from *Ecklonia cava,* (a) Extraction protocol and (b) High Performance Liquide Chromatography (HPLC) analysis of Dieckol.

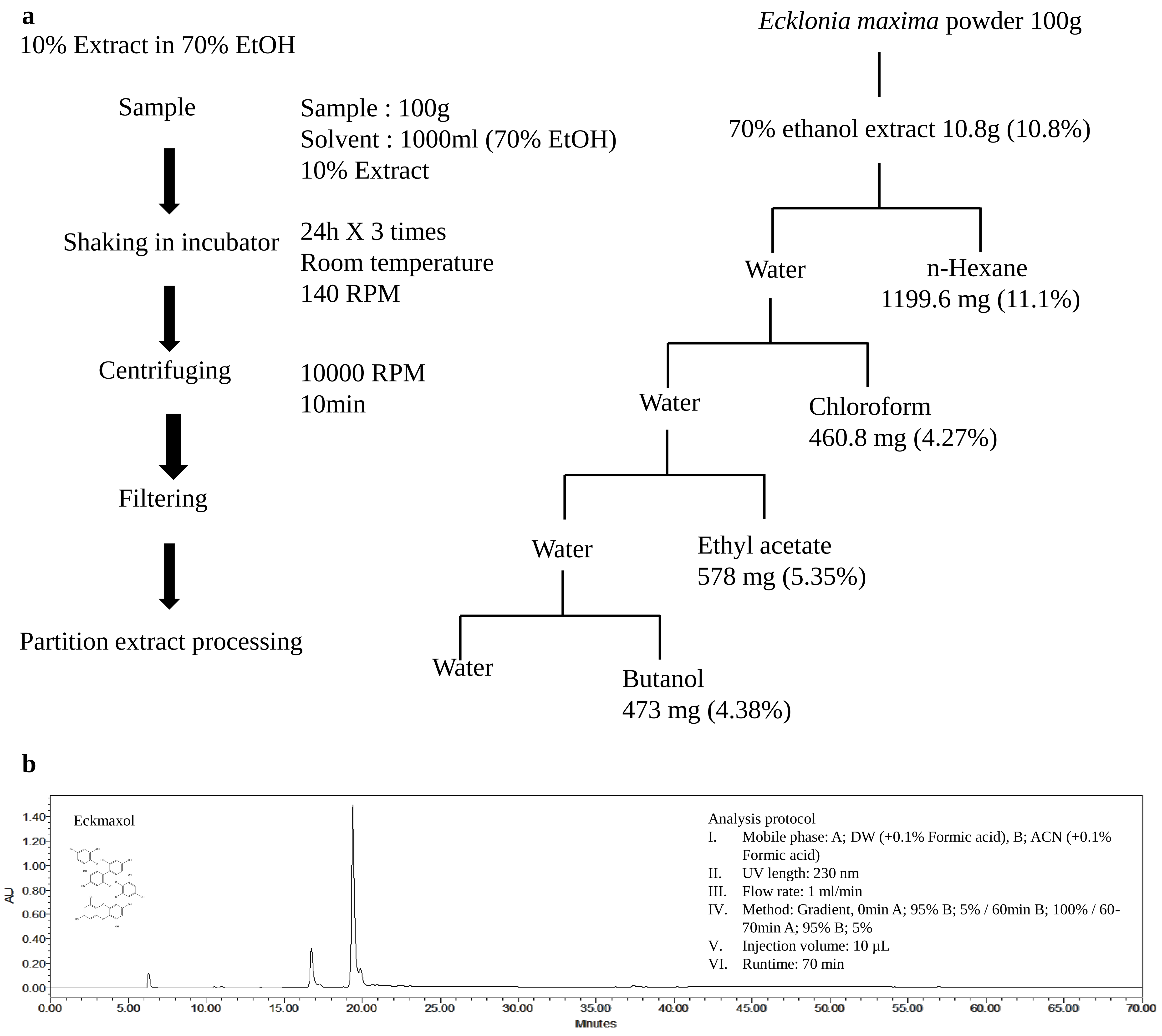

Supplementary Figure S13. Extraction and isolation procedure of Eckmaxol from *Ecklonia maxima*, (a) Extraction protocol and (b) High Performance Liquide Chromatography (HPLC) analysis of Eckmaxol.
